# Rosuvastatin attenuates airway inflammation and remodeling in a chronic allergic asthma model through modulation of the AMPKα signaling pathway

**DOI:** 10.1371/journal.pone.0305863

**Published:** 2024-06-24

**Authors:** Lei Zhang, Feng-Ying Huang, Shu-Zhen Dai, Lin Wang, Xiangdong Zhou, Zhen-You Zheng, Qi Li, Guang-Hong Tan, Cai-Chun Wang

**Affiliations:** 1 Department of Respiratory Medicine, The First Affiliated Hospital of Hainan Medical University & Hainan Province Clinical Medical Center of Respiratory Disease, Haikou, China; 2 NHC Key Laboratory of Tropical Disease Control, School of Tropical Medicine & The Second Affiliated Hospital, Hainan Medical University, Haikou, China; 3 Department of Ophthalmology, The First Affiliated Hospital of Hainan Medical University, Haikou, China; Mohammed Bin Rashid University of Medicine and Health Sciences, UNITED ARAB EMIRATES

## Abstract

The efficacy of rosuvastatin in reducing allergic inflammation has been established. However, its potential to reduce airway remodeling has yet to be explored. This study aimed to evaluate the efficacy of rosuvastatin in reducing airway inflammation and remodeling in a mouse model of chronic allergic asthma induced by sensitization and challenge with OVA. Histology of the lung tissue and the number of inflammatory cells in bronchoalveolar lavage fluid (BALF) showed a marked decrease in airway inflammation and remodeling in mice treated with rosuvastatin, as evidenced by a decrease in goblet cell hyperplasia, collagen deposition, and smooth muscle hypertrophy. Furthermore, levels of inflammatory cytokines, angiogenesis-related factors, and OVA-specific IgE in BALF, plasma, and serum were all reduced upon treatment with rosuvastatin. Western blotting was employed to detect AMPK expression, while immunohistochemistry staining was used to observe the expression of remodeling signaling proteins such as α-SMA, TGF-β, MMP-9, and p-AMPKα in the lungs. It was found that the activity of 5’-adenosine monophosphate-activated protein kinase alpha (AMPKα) was significantly lower in the lungs of OVA-induced asthmatic mice compared to Control mice. However, the administration of rosuvastatin increased the ratio of phosphorylated AMPK to total AMPKα, thus inhibiting the formation of new blood vessels, as indicated by CD31-positive staining mainly in the sub-epithelial region. These results indicate that rosuvastatin can effectively reduce airway inflammation and remodeling in mice with chronic allergic asthma caused by OVA, likely due to the reactivation of AMPKα and a decrease in angiogenesis.

## Introduction

Asthma is a complex condition with a global impact, affecting approximately 334 million people [1, 2]. It is a chronic condition marked by inflammation and remodeling of the airways, resulting in high medical costs, a decrease in quality of life, and, in severe cases, death if not treated with traditional treatments [3]. To improve asthma management, it is essential to gain an in-depth understanding of its pathophysiology and to search for more effective treatments.

Chronic allergic asthma is a condition characterized by an abnormal response of Th2 cells to allergens, resulting in a long-term inflammatory reaction and the remodeling of the airways [4, 5]. This remodeling includes the proliferation of mucosal cells, the accumulation of subepithelial fibrosis, an increase in airway smooth muscle mass and collagen deposition, and angiogenesis. Angiogenesis, which is supported by TGF-β, VEGF, and MMP-9 [6, 7], is particularly noteworthy, as it allows for an ongoing influx of inflammatory cells and mediators, as well as providing essential elements such as oxygen, nutrients, and growth factors for irregular cell growth and increased activity [8–10]. Bronchial angiogenesis is mainly dependent on VEGF and endothelial progenitor cells that are activated when stromal cell-derived factor 1 (SDF-1) binds to chemokine receptor CXCR4 [11, 12]. Therefore, an effective treatment of chronic allergic asthma should include a therapeutic approach that targets both the persistent airway inflammation and the remodeling of the airways.

Clinical studies have demonstrated that statins, a type of lipid-regulating medication, have a variety of non-lipid-lowering effects, such as immunomodulation, control of inflammatory reactions, antioxidative stress, and inhibition of mitosis in cancer cells [13–17]. Research conducted on asthmatic individuals has revealed that taking statins can be beneficial in terms of a decrease in asthma exacerbations, relieving symptoms, and lowering the risk of asthma-related hospitalizations and emergencies [18]. Furthermore, the daily cumulative dose of statins is inversely proportional to the frequency of asthma attacks [19, 20]. Clinical trials have also indicated that a combination of oral statin treatment and budesonide is effective in reducing lung inflammation in severely asthmatic patients by enhancing IL-10 production and suppressing macrophage autophagy [21]. Experimental studies have demonstrated that statins, such as rosuvastatin and simvastatin, can reduce inflammation [22]. Additionally, research has shown that when asthmatic mice are treated with rosuvastatin, the expression of eosinophils, neutrophils, and cytokines such as IL-4, IL-13, IL-5, and IL-17 is decreased, while the expression of the anti-inflammatory cytokine IL-10 is increased [22, 23]. This leads to a significant decrease in lung tissue inflammation and airway hyperresponsiveness. Moreover, it has been observed that systemic administration of simvastatin in a mouse model of allergic inflammation caused by mite antigens can reduce more than 50% of the lung inflammatory responses, including eosinophil and neutrophil infiltration, an increased number of hyperplastic goblet cells, and the secretion of pro-inflammatory cytokines [24]. Overall, the evidence from these studies supports the use of statins as a potential treatment option for asthma, as they have been shown to have anti-inflammatory and immunomodulatory effects that can provide relief for individuals with asthma.

AMP-activated protein kinase α (AMPKα), a significant signaling pathway related to cellular energy balance, has been observed to be a major regulator of physiological activities such as inflammation, metabolism, and autophagy [25–27]. Recent studies have suggested that activating AMPKα could have beneficial effects in asthma, such as inhibiting airway contraction, suppressing the activation and migration of inflammatory cells into the airways, reducing mucus production, and reducing oxidative stress [28, 29]. To further explore the anti-inflammatory effects of statins, this study will investigate whether rosuvastatin, a commonly used statin, can reduce asthma-related inflammation and airway remodeling through the AMPKα pathway.

## Materials and methods

### Ethics approval

All animal-based research studies have been conducted in compliance with the ARRIVE guidelines (https://arriveguidelines.org) and have been approved by the Ethics Committee for Animal Care and Use of Hainan Medical University (approval No. ETH_HMU201800128).

### Establishment of an ovalbumin-mediated mouse allergic chronic asthma model and treatment with rosuvastatin

**Fig 1:**
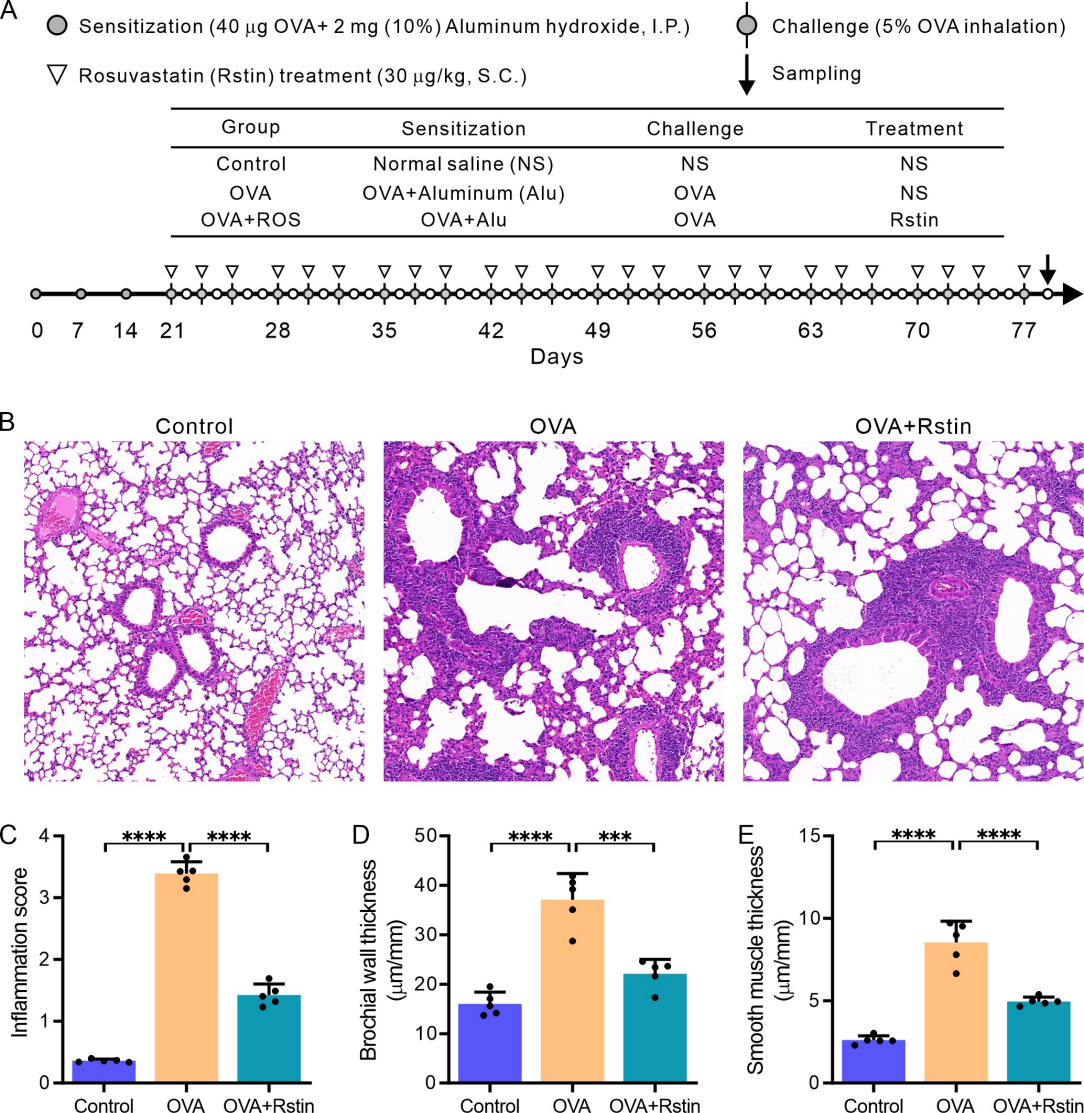
Rosuvastatin treatment on inflammatory infiltration in the lungs. (A) The illustration shows the establishment of the OVA-mediated chronic allergic asthma model in mice and the groups with or without PAC treatment. (B) The representative image of a lung tissue section stained with Hematoxylin and Eosin (HE) from each experimental group. (C) The blinded inflammation score obtained from the analysis of HE-stained sections. (C and D) The airway wall thickness (C) and smooth muscle thickness (D) from the HE-stained lung sections using Image-Pro Plus software. All images were captured at a magnification of ×200. The data are expressed as the mean ± SD (n = 5) and analyzed using one-way ANOVA followed by Tukey’s post-hoc test. Statistical significance is denoted as *P < 0.05, **P < 0.01, ***P < 0.001, and ****P < 0.0001.

Mice aged between 6–8 weeks were acquired from the Chengdu Animal Center (Sichuan, China) and kept in a temperature-regulated, pathogen-free atmosphere with a 12-hour light-dark cycle. Thirty mice were randomly allocated into three separate groups: the Control, the OVA, and the OVA+Rstin groups. All the groups were given an ample supply of standard OVA-free food and water. The chronic allergic mouse asthma model was established based on a previously documented protocol [30], with a minor adjustment to prolong the study to 78 days, as illustrated in Fig 1A. In brief, mice were administered a total of 40 μg of OVA (Grade V, Sigma Aldrich, St. Louis, MO) along with 2 mg of aluminum hydroxide (ImjectAlum; Pierce Biotechnology, Rockford, IL) in a volume of 200 μL through intraperitoneal injection on Days 0, 7, and 14. From the 21st day onwards, the OVA-sensitized mice were subjected to an eight-week period of exposure to 5% OVA (Grade V, Sigma Aldrich) aerosol in a sealed environment, facilitated by a nebulizer (PARI Respiratory Equipment, Richmond, VA). The exposure was carried out five times a week, with each session lasting 30 minutes. The OVA+Rstin group of mice were administered a subcutaneous injection of rosuvastatin (30 mg/kg, Sigma-Aldrich) 30 minutes prior to each inhalation challenge on the same day, whereas the OVA group did not receive the rosuvastatin treatment [31]. The Control group followed the same protocol; however, they were administered saline instead of OVA for sensitization and challenge, and rosuvastatin. Upon completion of the last experiment, all mice were humanely euthanized according to previously reported methods [32], which involved an intraperitoneal injection of a combination of Ketamine (250 mg/kg) and Xylazine (25 mg/kg) until the toe pinch and corneal reflex were no longer present. Subsequently, BALF, blood, and lung tissues were collected by an intraperitoneal injection of an excessive amount of pentobarbital sodium (illustrated in Fig 1A).

### Lung histopathology

Following the collection of BALF, the left lung was fixed in 4% formalin solution for 24 hours before being embedded in paraffin wax. The right lung was stored in liquid nitrogen for future Western blot analysis. To assess the presence of inflammatory cell infiltrations, mucus secretions, and subepithelial fibrosis, sections (5 μm) of the paraffin-embedded lung tissue were stained with Hematoxylin and Eosin (H&E), Periodic Acid-Schiff (PAS), and Masson’s Trichrome dyes, respectively. A Nikon ECLIPSE 80i light microscope (Tokyo, Japan) at 40x magnification was employed to measure the inner diameters of 20 randomly chosen bronchioles, which ranged from 100 to 200 μm. A double-blind, quantitative analysis of the lung tissues was conducted by two independent experimenters using Image-Pro Plus v6.0 software (Media Cybernetics, Rockville, MD). A five-point scale was used to evaluate the degree of inflammation of the airway wall and smooth muscle layers, as well as the mucus score based on the percentage of goblet cells found in the epithelium [33]. Furthermore, Masson’s Trichrome staining was used to measure the degree of collagen deposition in the submucosal region and the basement membrane. The area of Masson’s staining per unit length of the basement membrane (μm) was calculated to quantify the results [34].

### Immunohistochemistry of lungs

Lung immunohistochemistry was performed as previously reported [35]. In brief, the lung tissue sections were processed by dewaxing and rehydrating them with a xylene and ethanol solution. Subsequently, a 10 mmol/L sodium citrate buffer was applied using a microwave to retrieve the antigens, and 3% hydrogen peroxide was used to inhibit the endogenous peroxidase. This was followed by a 30-minute blocking step with 3% bovine serum albumin. The sections were then incubated with primary antibodies, including anti-α-SMA (1:1500), anti-MMP-9 (1:300), anti-TGF-β (1:450), anti-CD31 (1:200), and anti-p-AMPKα (Thr172, 1:200), purchased from Servicebio (Wuhan, China) or Cell Signaling Technology (Danvers, MA, United States). After an overnight incubation at 4°C, these sections were labeled with either goat anti-rabbit or mouse antibodies, and the DAB Kit (Abcam ab64238, Shanghai, China) was used to visualize them. Finally, the intensity of the brown color in the peribronchial area was quantitatively assessed using Image-Pro Plus software (Media Cybernetics, Rockville, MD, USA).

### Immunofluorescence detection of lung sections

Immunofluorescence detection of lung sections was performed in accordance with a previously reported method [36]. To begin, the lung tissue sections were permeabilized with 0.1% Triton X-100 and blocked with 3% bovine serum albumin. Afterwards, the sections were incubated with a rabbit anti-CD31 antibody (ab124432; Abcam, Shanghai, China) at a dilution of 1:100 overnight at 4°C. Afterwards, a FITC-labeled anti-rabbit IgG secondary antibody (1:300; ab313801, Abcam) was applied to the lung slices for an hour at room temperature to complete the staining process. Following the washing, the lung slices were placed in a medium containing DAPI for mounting. Finally, using a fluorescence microscope and a digital photomicrograph system (LX83, Olympus, Tokyo, Japan), the green fluorescent images were captured and quantified objectively using free ImageJ software.

### Western blotting analysis

Frozen lung tissue samples of approximately 100 mg were homogenized in 1,000 μL of RIPA buffer containing protease inhibitors obtained from Boster Biotech (Wuhan, China), and the homogenate was then subjected to Western blotting analysis as previously described [37]. The lysates were then centrifuged at a speed of 12,000 rpm for 20 minutes at a temperature of 4°C to separate the proteins. Following the detection of the protein concentration of the supernatant using a BCA Protein Assay Kit (Takara, Dalian, China), the proteins were then separated via 12% sodium dodecyl sulfate polyacrylamide gel electrophoresis and transferred onto PVDF membranes using a Mini Trans-Blot Protein Transfer System (Bio-Rad, Hercules, CA, USA). The membranes were then treated with a 5% skim milk solution for one hour to block them, and were subsequently incubated with primary antibodies against AMPKα (ab32047; Abcam) at a dilution of 1:1,500 and p-AMPKα (Thr183 and Thr172) (ab133448; Abcam) at a dilution of 1:2,000. Following this, the HRP-conjugated anti-IgG antibodies were left to incubate the lung slices at room temperature for an hour, in order to detect the primary antibodies. Finally, the blotted lung sections were incubated with an enhanced chemiluminescence substrate for 30 minutes, and then the images were obtained by a UVP ChemStudio system (Analytik Jena, Upland, CA). This was subsequently quantified using ImageJ software.

### Determination of the inflammatory cell subtypes and cytokines in BALF

The BALF was examined for interleukins and inflammatory cells, such as total inflammatory cells, eosinophils, lymphocytes, and neutrophils, using standard protocols [38]. In brief, the BALF was centrifuged at a rate of 170 g for 10 minutes, and the cell pellet was then resuspended in 1 mL of PBS. To ascertain the total cell count, 90 μL of cell suspensions were stained with 10 μL of 0.2% Crystal Violet. The quantification of eosinophils, lymphocytes, and neutrophils was carried out according to criteria that had been previously established [38]. In addition, the supernatants of the BALF were collected, and the concentrations of L-4, IL-13, IL-5, TGF-β1, TNF-α, and MMP-9 were determined and quantified using ELISA kits (Boster, Wuhan, China) as per the instructions provided by the manufacturer. The Optical Density (OD) of the solution was then determined using an ELISA reader (ELX808IU, Bio-Tek, VT) at 450 nm. A standard preparation of known concentrations was used to create a standard concentration curve, which allowed the calculation of the concentrations of the samples from their OD values.

### Detection of OVA specific IgE and cytokines in blood

Samples of blood were taken by extracting from the eyes and divided into pyrogen-free and EDTA-anticoagulated tubes to collect the serum, as mentioned before [35]. Thereafter, the concentration of OVA-specific IgE, CXCR4, VEGF, and SDF-1 in the serum was measured using ELISA kits (Boster, Wuhan, China) as per the manufacturer’s instructions. An ELISA reader (ELX808IU, Bio-Tek, VT) was then utilized to read the ELISA plates at 450 nm. A concentration curve was constructed and the concentrations of the IgE and cytokines were determined based on the OD values of the samples.

### Statistical analysis

The mean and standard deviation (SD) of the data were reported. A one-way analysis of variance (ANOVA) was employed, followed by Tukey’s post hoc test, and the results were analyzed using GraphPad Prism v9.0.0 software (GraphPad, San Diego, CA). Statistical significance was established at P < 0.05 (*), P < 0.01 (**), P < 0.001 (***), and P < 0.0001 (****) with adjusted P-values.

## Results

### Rosuvastatin treatment reduces airway inflammation and remodeling in mice with chronic allergic asthma

Histopathological analysis of H&E-stained slides demonstrated that, in comparison to the Control group, which was administered a normal saline solution, mice exposed to OVA had an increased presence of inflammatory cells near the bronchi in the pulmonary parenchyma. However, this inflammatory cellular infiltration was significantly reduced in mice that were administered rosuvastatin (Fig 1B). Subsequently, a significant decrease in the inflammatory score, derived from peribranchial inflammatory cells, was observed in the OVA+Rstin group compared to the OVA group (Fig 1C). Additionally, the OVA group, which had been exposed to OVA for an extended period of time, had an increased bronchial wall thickness (Fig 1D) and smooth muscle thickness (Fig 1E) compared to the Control group of mice; yet, the OVA+Rstin group that was treated with rosuvastatin experienced a decrease in these modifications. These findings demonstrate the successful induction of airway inflammation and remodeling in the chronic allergic asthma model mice, caused by sensitization and recurrent challenge with OVA, as well as the efficacy of rosuvastatin treatment in alleviating these histological changes.

### Rosuvastatin treatment attenuates airway remodeling in the histological examination of the lungs of OVA-mediated chronic allergic mice

**Fig 2:**
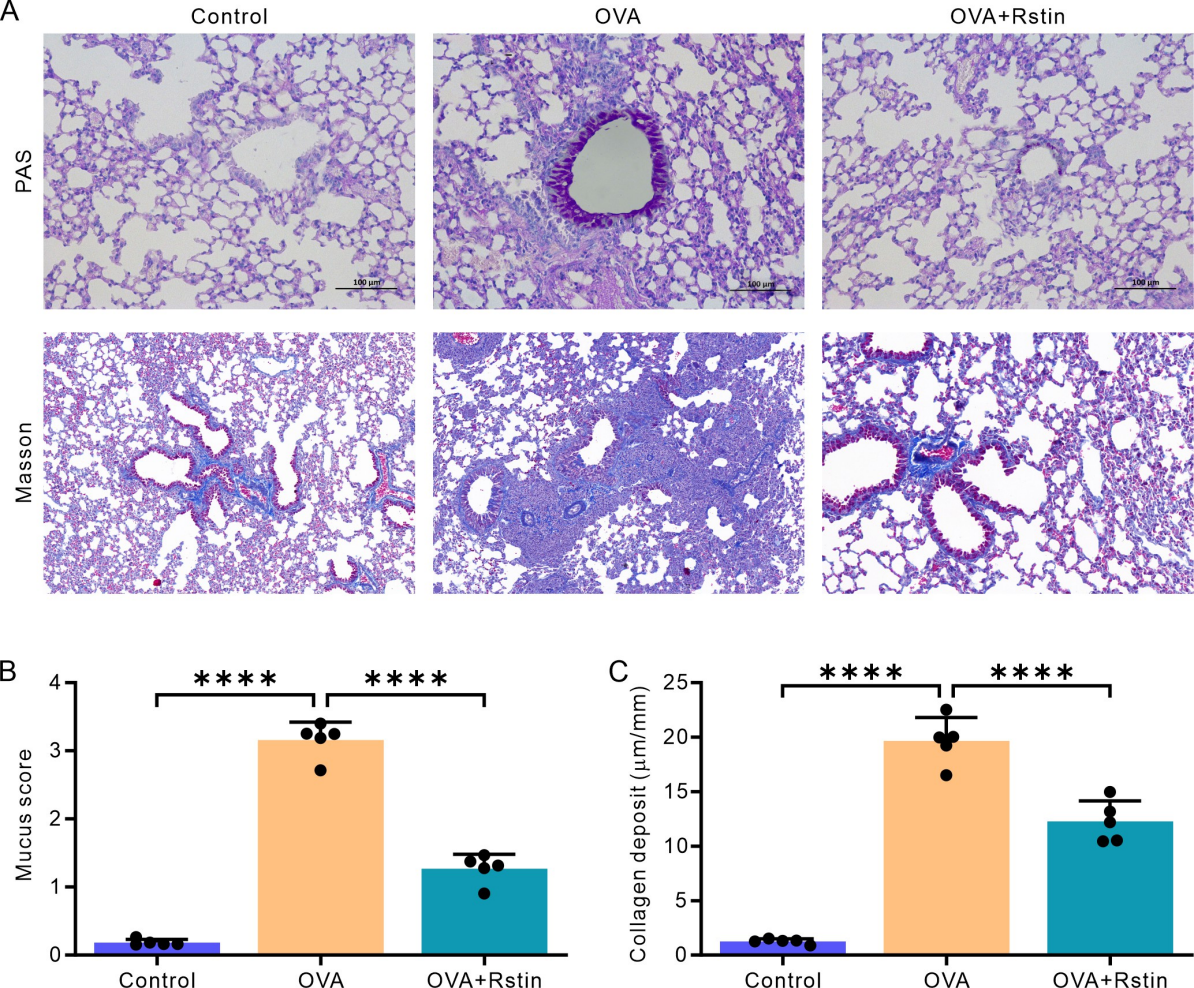
Rosuvastatin treatment mitigates airway remodeling in the histology. (A) Representative images of lung sections stained with PAS (upper) and Masson’s trichrome (down) from each group. (B) Blinded scoring of the percentage of PAS-positive epithelial cells. (C) Blinded quantitative analyses of the area of peribronchial Masson’s trichrome staining using Image-Pro Plus. All images were captured at a magnification of ×200. The data are presented as the mean ± SD (n = 5) and were analyzed using one-way ANOVA followed by Tukey’s post-hoc test. Statistical significance is indicated as *P < 0.05, **P < 0.01, ***P < 0.001, and ****P < 0.0001.

**Fig 3:**
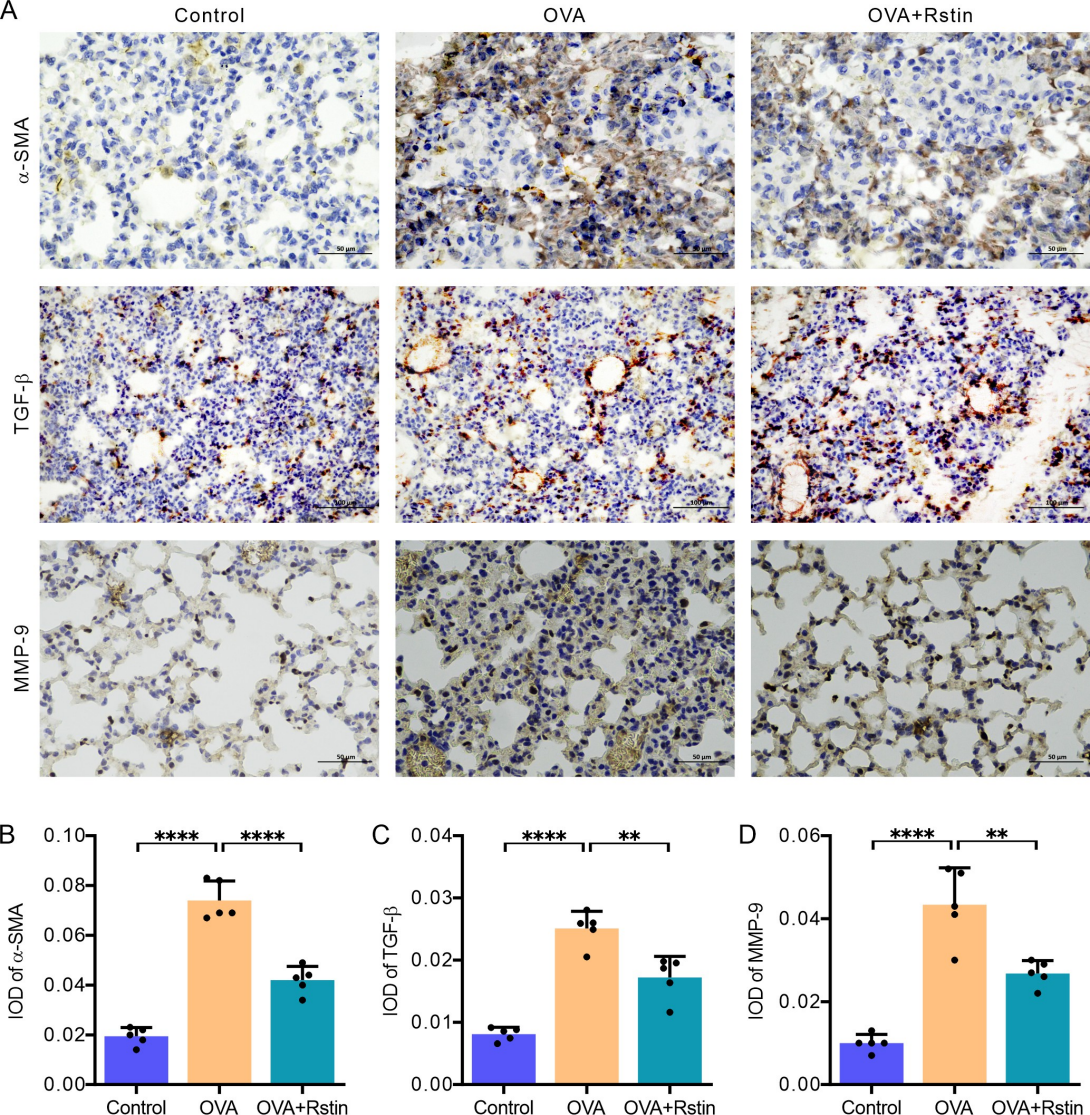
Rosuvastatin treatment exhibits anti-fibrotic effects. (A) Representative images displaying the immunoreactivity of α-SMA, TGF-β, and MMP-9 in lung tissues. (B–D) Quantitative analysis of α-SMA (B), TGF-β (C), and MMP-9 (D) immunohistochemical integral optical density (IOD) using Image-Pro Plus. The images were captured at a magnification of ×200. The data is presented as the mean ± SD (n = 5) and was analyzed using one-way ANOVA, followed by Tukey’s post-hoc test. Statistical significance is denoted as *P < 0.05, **P < 0.01, ***P < 0.001, and ****P < 0.0001.

In this study, we employed PAS, Masson’s Trichrome, and immunohistochemistry staining techniques to identify and quantify airway remodeling features, such as mucus production in the bronchi, collagen accumulation in the vicinity of the bronchi, proliferation of airway smooth muscle cells, and airway fibrosis. Analysis of PAS-stained lung sections revealed a notable hyperplasia of goblet cells in the OVA group, which was ameliorated by rosuvastatin treatment (Fig 2A). This was further confirmed by the mucus score, which was significantly higher in the OVA group compared to the Control group; and, as expected, administration of rosuvastatin resulted in a significant decrease in the mucus score in the mice from the OVA+Rstin group (Fig 2B). As anticipated, Masson’s staining demonstrated that sensitization and recurrent challenge with OVA resulted in increased marker collagen deposition around the bronchi in the mice from the OVA group, yet, rosuvastatin treatment significantly decreased this deposition (Fig 2A and 2C). In addition, immunohistochemistry staining of alpha-smooth muscle actin (α-SMA) showed an increase in α-SMA immunostaining in the chronic OVA exposure group compared to the Control group, which was reversed by rosuvastatin treatment (Fig 3A and 3B). TGF-β and MMP-9 are proteins associated with fibrosis and indicate airway remodeling. The immunohistochemistry analysis revealed that the expression of both TGF-β and MMP-9 was significantly higher in the recurrent OVA-challenged mice from the OVA group when compared to the Control mice only treated with saline; however, the expression of these two proteins was significantly decreased in the mice from the OVA+Rstin group (Fig 3A, 3C and 3D). Taken together, these findings demonstrate that rosuvastatin is capable of diminishing the prominent characteristics of the airway remodeling in this experimental chronic allergic asthma model.

### Effect of rosuvastatin treatment on cytokine production, IgE levels, and angiogenesis in the lungs of OVA-mediated chronic allergic mice

**Fig 4:**
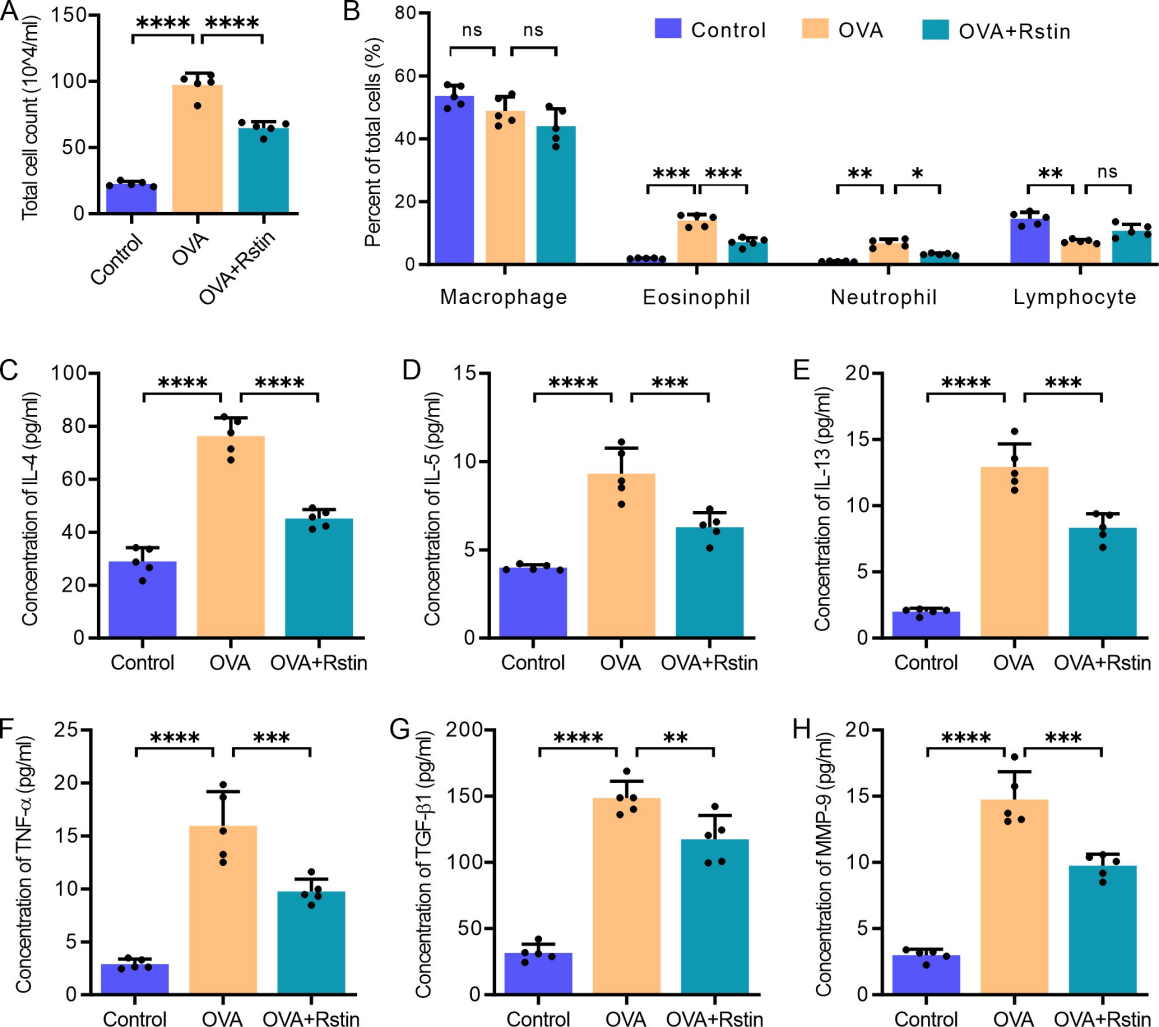
Rosuvastatin treatment reduces the concentrations of various cytokines in the BALF. (A and B) Comparison of total cell numbers (A) and differential cell counts (B) in the BALF. (C-H) The concentrations of cytokines, including IL-4 (C), IL-5 (D), IL-13 (E), TNF-α (F), TGF-β (G), and MMP-9 (H) in the BALF, were detected by ELISA. The data were presented as the mean ± standard deviation (n = 5) and were analyzed using one-way analysis of variance (ANOVA), followed by Tukey’s post-hoc test. Statistical significance is denoted as *P < 0.05, **P < 0.01, ***P < 0.001, and ****P < 0.0001.

**Fig 5:**
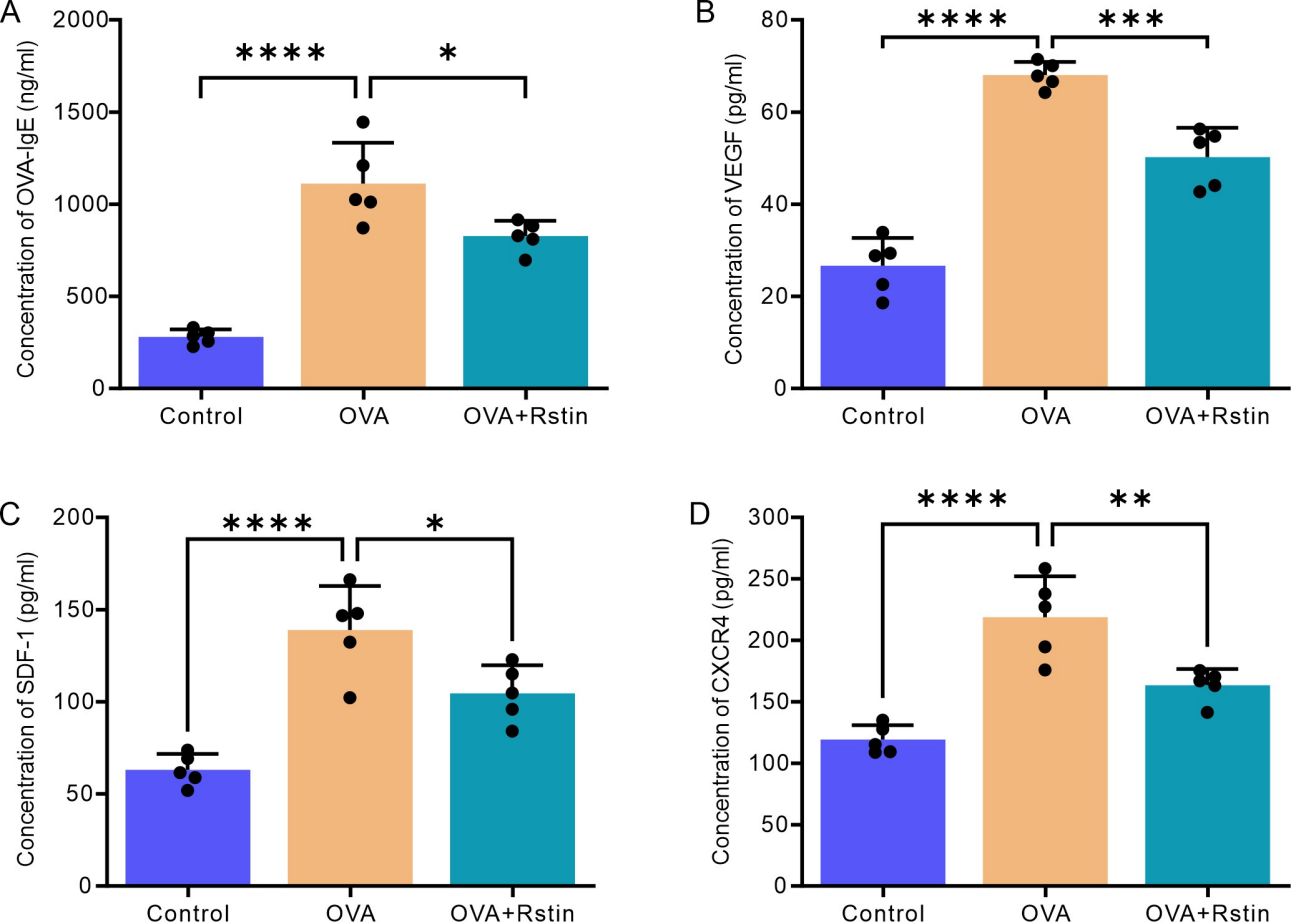
Rosuvastatin treatment decreases the levels of OVA-IgE and angiogenesis-related cytokines in the serum. (A-D) The concentrations of OVA-specific IgE (A), VEGF (B), SDF-1 (C), and CXCR4 (D) in the serum were detected by ELISA. The data were presented as the mean ± standard deviation (n = 5) and were subjected to one-way analysis of variance (ANOVA), followed by Tukey’s post-hoc test. Statistical significance is denoted as *P < 0.05, **P < 0.01, ***P < 0.001, and ****P < 0.0001.

The level of inflammation in the lung tissues was evaluated by assessing the number of macrophages, neutrophils, eosinophils, and lymphocytes, as well as by analyzing the concentrations of cytokines in the BALF and the serum. In comparison to the mice from the Control group only treated with normal saline, the total cell counts of inflammatory cells and the percentages of eosinophils and neutrophils in the OVA group increased after two months of recurrent exposure to OVA; however, administration of rosuvastatin was successful in decreasing the total cell count and the percentages of eosinophils and neutrophils in the BALF (Fig 4A and 4B). Additionally, the ELISA results revealed a significant increase in the concentrations of Th2 cytokines, including IL-4 (Fig 4C), IL-5 (Fig 5D) and IL-13 (Fig 4E) in the BALF of mice from the OVA group sensitized and challenged with OVA, compared to the mice from the Control group treated with saline; however, the concentrations of these cytokines were substantially decreased in the mice treated with rosuvastatin in the OVA+Rstin group. Similarly, the three remodeling characteristic proteins, including TGF-β (Fig 4F), TNF-α (Fig 4G) and MMP-9 (Fig 4H) in the BALF were also found to be increased in the mice from the OVA group, but rosuvastatin reversed their concentrations. Moreover, analysis of blood samples using ELISA revealed that levels of OVA-specific IgE (OVA-IgE) were significantly increased in the serum of mice with OVA-induced chronic allergic asthma compared to those in the Control group that had been treated with saline only. Furthermore, angiogenesis-associated factors such as VEGF (Fig 5B), SDF-1 (Fig 5C), and CXCR4 (Fig 5D) were also significantly elevated in the serum of the OVA-induced allergic asthma mice. However, these effects were significantly reduced in mice from the OVA+Rstin group (Fig 5B–5D). Collectively, these findings suggest that rosuvastatin has an inhibitory effect on the Th2 immune response and the generation of numerous proinflammatory, pro-fibrotic, and pro-angiogenic substances.

### Rosuvastatin restores AMPKα activity and suppresses angiogenesis in the lungs of OVA-mediated allergic mice

**Fig 6:**
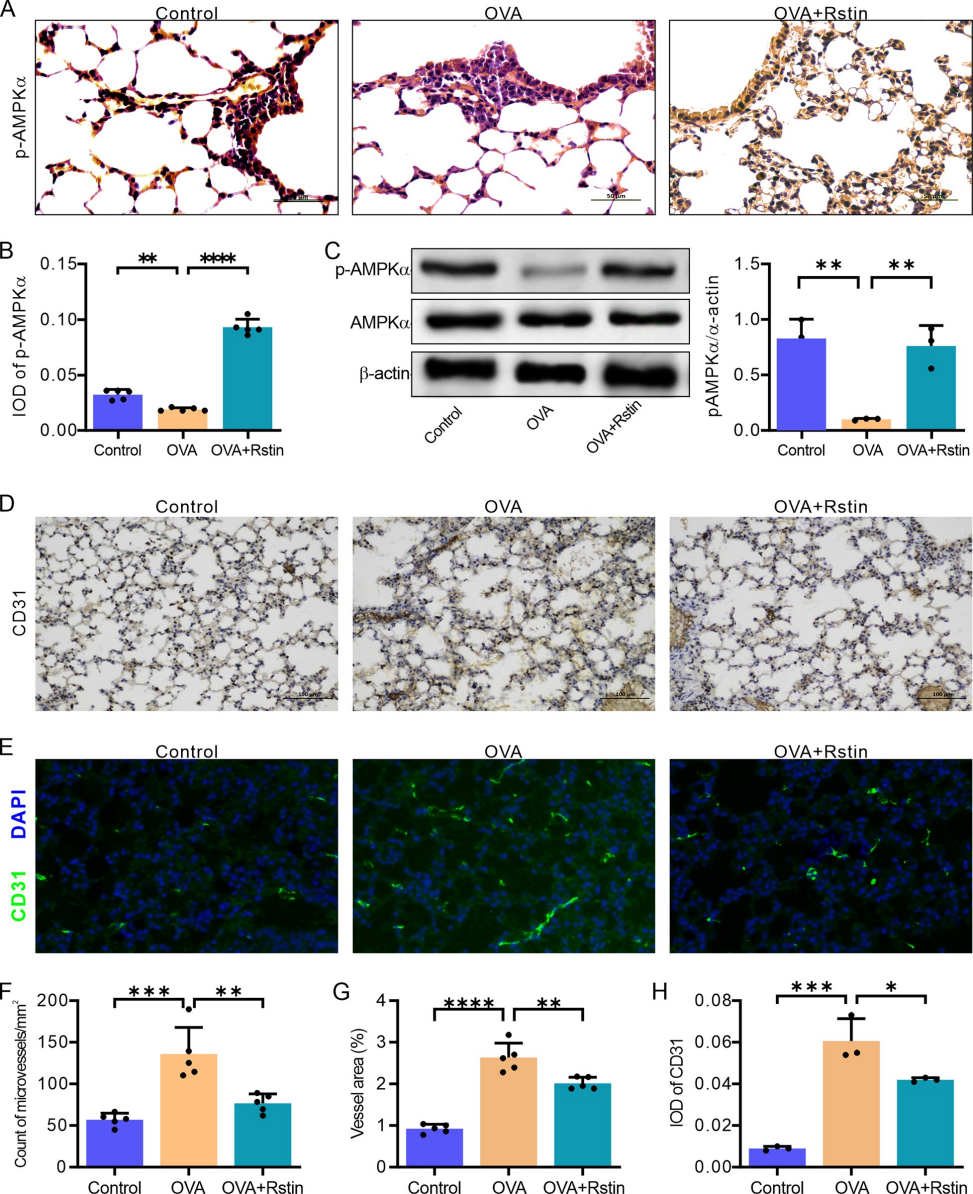
Effects of rosuvastatin treatment on AMPK activity and angiogenesis. (A and B) Representative images (A) and blinded quantitative analyses (B) of p-AMPK levels detected by immunohistochemistry in lung sections. (C) Western blotting and densitometric analyses of p-AMPK and AMPK in lung tissues. (D-G) Immunohistochemical (D) and immunofluorescent (E) staining of lung sections, showing the presence of microvessels, which were further quantified using blinded quantitative analysis of the number (F) and area (G) of microvessels positive for CD31 immunostaining in the sub-epithelial region as well as fluorescence intensities for CD31 staining in the sub-epithelial region (H). Statistical analysis using one-way ANOVA, followed by Tukey’s post-hoc test showed that the results were statistically significant (P < 0.05, P < 0.01, P < 0.001, and P < 0.0001).

Immunohistochemical staining and Western blotting were used to assess the levels of AMPKα and its phosphorylated form, p-AMPKα (Thr172), in lung tissue in order to understand the effects of rosuvastatin. The p-AMPKα staining revealed a significant presence of p-AMPKα-positive cells in the recurrent OVA-challenged allergic asthmatic mice treated with rosuvastatin, which was observed in almost every cell when compared to the Control and OVA groups (Fig 6A). This was quantified as integral optical density (IOD) and showed an approximate two-fold increase in the mice treated with rosuvastatin compared to the Control group, and above four-fold increase compared to the OVA group (Fig 6B). Consistently, analysis by Western blotting showed that p-AMPKα expression was significantly lower in the OVA group than in the Control group, but it was substantially higher in the OVA+Rstin group (Fig 6C). Surprisingly, the expression of AMPKα remained unchanged across the three groups (Fig 6C). These findings indicate that rosuvastatin could amplify the expression of both AMPKα and its phosphorylated form, p-AMPKα, in epithelial and endothelial cells in the lungs of mice with chronic allergic asthma caused by OVA exposure. Furthermore, we investigated the relationship between the activation of AMPKα and angiogenesis in the subepithelium of mice with OVA-induced chronic allergic asthma. Additionally, immunohistochemistry analysis using a monoclonal antibody against CD31, a marker of angiogenesis, revealed that, in comparison to Control mice, OVA-induced chronic allergic mice had a greater number (Fig 6D and 6F) and area (Fig 6D and 6G) of microvessels in the subepithelium; however, rosuvastatin treatment significantly reduced these increases. Consistently, immunofluorescence quantitative analysis of CD31-positive green signal in the airway also exhibited a noticeable decrease in the rosuvastatin-treated mice compared to the OVA-induced chronic allergic mice (Fig 6E and 6H). These results suggest that the activation of AMPKα may inhibit angiogenesis in the vicinity of the bronchioles.

## Discussion

In this study, our research has demonstrated that rosuvastatin treatment can be used to effectively reduce airway inflammation and remodeling in a chronic asthma mouse model caused by sensitization to OVA and inhalation. Through animal experiments, we observed that rosuvastatin treatment was successful in decreasing the pathological changes in the lungs, such as the accumulation of inflammatory cells, the outflow of inflammatory mediators, hyperplasia of goblet cells, hypertrophy of the airway smooth muscle, subepithelial fibrosis, and neovascularization. Furthermore, we discovered that AMPKα activity was deficient in the mouse airways due to long-term OVA exposure, and that this was restored by rosuvastatin treatment.

Our results are consistent with the evidence that statins can offer protection from pulmonary inflammation in asthmatic mice [21, 24, 39]. Nevertheless, these studies only looked at the short-term effects of statins, either as monotherapy or combination therapy, and were conducted using mite-induced allergic inflammation mouse models. As asthma is a chronic disorder characterized by persistent inflammation and remodeling of the airways, a more precise evaluation of the long-term effectiveness of rosuvastatin requires a longer intervention. Therefore, we employed a mouse model of chronic allergic asthma, induced by a two-month OVA inhalation challenge, to examine the particular effects of rosuvastatin. We consider that the mouse model of chronic allergic asthma constructed in this study is more suitable for investigating airway remodeling in mice.

In this study, we employed a variety of histological techniques, such as PAS, Masson, and α-SMA staining, to analyze lung tissue sections. Our results showed that rosuvastatin treatment can reduce the number of goblet cells that proliferate and secrete mucus, as well as significantly reduce collagen deposition in the subepithelial layer. Additionally, it was found to increase the generation of ASM mass. This finding is in line with the results of Zeki et al., who found that statins can effectively alter multiple key indicator molecules of airway remodeling and improve airway remodeling in a rat model of chronic airway inflammation induced by ovalbumin and fungal allergen protease [40]. Additionally, previous research has demonstrated that statin therapy may be effective in inhibiting the growth and spread of airway smooth muscle cells when exposed to various stimuli in vitro. Additionally, experiments conducted on mouse models of pulmonary fibrosis have provided evidence that statin therapy can be beneficial in preventing the progression of the condition [41–43]. Given that airway remodeling is a common feature of chronic inflammatory lung diseases such as asthma and COPD, in combination with our current findings, it suggests that statins may be a viable option for treating these chronic lung inflammations with pulmonary fibrosis and remodeling.

The extracellular matrix (ECM) is a three-dimensional structure of molecules, including proteins, polysaccharides, and other biological molecules, that is located outside the cell [44]. This complex arrangement is of immense importance, as it provides structural support, enables cell movement, and assists in the transmission of signals [44]. To further validate the anti-fibrotic properties of rosuvastatin treatment in asthma, we investigated two significant remodeling mediators: TGF-β and MMP-9. In this study, immunohistochemistry and ELISA were employed to assess the levels of these molecules in lung tissue following rosuvastatin treatment. It was observed that rosuvastatin treatment significantly decreased the expression of TGF-β and MMP-9. Additionally, immunohistochemistry and immunofluorescence results revealed that rosuvastatin inhibited the formation of new blood vessels in the subepithelial region of chronic allergic asthma mice, thereby reducing the infiltration of inflammatory cells and mediators, and improving the composition and structure of the extracellular matrix. These findings suggest that rosuvastatin may be an effective therapeutic agent in controlling and improving airway remodeling in chronic allergic asthma, as excessive accumulation or degradation of the ECM can lead to the proliferation and fibrosis of the ECM in chronic allergic asthma.

In this study, our findings demonstrate the effectiveness of rosuvastatin in reducing the presence of eosinophils and neutrophils, as well as the production of Th2 cytokines and OVA-specific IgE in a mouse model of chronic asthma caused by OVA, which is a characteristic of Th2-driven inflammation. This is in agreement with the results of prior studies on OVA-mediated chronic asthma models [13], and further indicates that rosuvastatin treatment leads to a significant decrease of not only Th2 cytokines, but also other mediators, including TNF-α, MMP9, and TGF-β1. This is also in accordance with other disease models, where lovastatin treatment has been found to inhibit the upregulation of TGF-β1 and TNF-α by focusing on several cell signaling pathways [23, 43, 45]. Additionally, we found that the administration of rosuvastatin was effective in decreasing the levels of VEGF, SDF-1, and CXCR4 in the serum of mice with chronic allergic asthma. This could hypothetically lead to a decreased formation of pulmonary neovascularization and a decrease of inflammatory mediators in asthma. This result is consistent with earlier research in non-asthma models, which showed that rosuvastatin treatment could effectively lower VEGF expression and impede the migration of endothelial progenitor cells [46, 47]. However, the precise mechanism by which rosuvastatin therapy affects asthma angiogenesis, matrix cytokines, and other biological mediators is still not known, and further research is required. Taken together, these results imply that rosuvastatin has a strong anti-inflammatory effect in chronic allergic asthma, and its mechanism of action is more intricate than merely suppressing the Th2 immune response.

In order to gain further insight into the impacts of rosuvastatin on the symptoms of chronic asthma, we conducted an analysis of the frequency and activity of AMPKα. In this study, results from immunohistochemical analysis revealed that the active phosphorylated form of AMPKα (p-AMPKα) had significant expression in the vascular endothelial and epithelial cells of the Control mice compared to those from the chronic allergic mice sensitized and recurrently challenged with OVA. However, rosuvastatin treatment resulted in a noteworthy increase in AMPKα activity, evidenced by a significant expression of p-AMPKα observed in the chronic allergic mice induced by OVA sensitization and recurrent challenge. This finding is consistent with the results of Wang et al., who reported that mouse lungs exposed to OVA combined with FAP exhibited higher concentrations of both total AMPKα and p-AMPKα [48]. At present, it has been suggested that the AMPK α1 and α2 subtypes are necessary for the maintenance of pulmonary function of endothelial and epithelial barriers [49]. A decrease in AMPK α2 activity has been linked to damage of airway epithelia and vascular endothelia [50]. Our study findings suggest that rosuvastatin may be an effective treatment for chronic allergic asthma inflammation and the inhibition of airway fibrosis remodeling, due to its ability to induce active expression of p-AMPKα.

This study was restricted to employing a mouse model of chronic allergic asthma, which only permitting us to observe the advantages of rosuvastatin treatment on chronic allergic inflammation at the whole-animal level. Moreover, there was a flaw in the design of our research project. In our current study, we divided the experimental mice into three groups: the first group served as the control group, where the mice did not undergo OVA induction to develop chronic asthma inflammation and only received normal saline (NS) throughout the experiment; the second group was the OVA-induced inflammation group, where the same NS was used to dissolve the OVA; and the third group was the Rosuvastatin treatment group, where Rosuvastatin was administered to the mice along with OVA induction in the second group. We believed that including a group of control mice treated with Rosuvastatin to observe the potential side effects of Rosuvastatin on the control mice could improve the results of our study and reinforce our findings and conclusions. Overall, in order to better understand how rosuvastatin treatment impacts the activity of the inflammatory immune microenvironment, as well as the complex factors of the extracellular matrix, the multiple cell types in chronic allergic asthma, and its upstream and downstream target pathways, a more comprehensive investigation using in vitro models is needed. Furthermore, because chronic allergic animal models and in vitro cell models can only partially replicate the pathological changes that occur in human chronic allergic asthma patients, further research incorporating samples and data from clinical allergic asthma patients is urgently needed.

Statins work by inhibiting the enzyme HMG-CoA reductase, which is involved in cholesterol synthesis. Recent studies have shown that statins also have anti-inflammatory properties, reduce cytokine levels, lower oxidative stress, inhibit myofibroblast differentiation, prevent lung tissue remodeling, and hinder processes associated with fibrosis. These medications have been found to directly affect macrophages and cytokines, leading to anti-fibrotic effects [51]. Research has indicated that simvastatin can reduce collagen deposition in alveolar septa, thus decreasing the development of pulmonary fibrosis in mice [52]. An analysis of a population-based cohort revealed that statin use is independently associated with a lower risk of interstitial lung disease and idiopathic pulmonary fibrosis in a dose-dependent manner [53]. Chronic asthma involves airway remodeling, characterized by thickening of airway walls, an increase in smooth muscle mass, changes in the extracellular matrix, and abnormalities in pulmonary interstitial angiogenesis. These changes are thought to contribute to the long-term decline in lung function and the development of irreversible airflow obstruction [54]. In our study, we primarily used immunohistochemical methods to evaluate the effects of rosuvastatin on OVA-induced pulmonary remodeling in mice by examining α-SMA, TGF-β, and MMP9. Our results are consistent with these studies, showing that rosuvastatin effectively inhibits pulmonary fibrosis in mice with OVA-induced allergic asthma. Moreover, recent studies indicate that statins have a dose-dependent, biphasic effect on angiogenesis [55]. They promote angiogenesis at low doses, but inhibit it at higher doses. Although there is no definitive evidence on the effects of high doses of statins in mice, the typical dose used in current research is approximately 10–15 mg/kg [55]. In our study, we administered a dose of 30 mg/kg of rosuvastatin to mice, which is considered a high dose. Through the use of immunohistochemistry and immunofluorescence, we observed that this high dose of rosuvastatin significantly suppressed angiogenesis in the lungs of mice with OVA-induced allergic asthma. Additionally, studies have suggested that statin treatment can decrease the expression of adhesion molecules in endometrial cells, potentially reducing the infiltration of immune cells into the lungs [56, 57]. This could be one of the mechanisms by which rosuvastatin regulates chronic allergic asthma. In summary, our current data, together with previous study results, indicate that the molecular mechanisms by which statins regulate chronic asthma inflammation and airway reconstruction are intricate. While our study provides mainly observational findings, further research is necessary to fully comprehend the underlying mechanisms. It is crucial to conduct more comprehensive experiments to explore how rosuvastatin inhibits chronic asthma inflammation and airway remodeling.

## Conclusions

Our research has shown the potential of rosuvastatin to treat a chronic airway inflammation and pulmonary fibrosis-associated remodeling in a mouse model of OVA-induced chronic allergic asthma. This is likely due to the activation of AMPKα signaling pathway, which leads to the inhibition of angiogenesis and a shift in the accumulation of ECM cytokines and mediators that are beneficial in reducing airway inflammation. Thus, our findings suggest an alternate therapeutic approach for treating chronic allergic asthma.

## Supporting information

S1 Raw images (PDF)

S1 Data (PDF)

## Abbreviations

AMPK: Adenosine Monophosphate Activated Protein Kinase
BALF: Bronchoalveolar lavage fluid
COPD: Chronic obstructive pulmonary disease
DAPI: 4’,6-diamidino-2-phenylindole
ECM: Extracellular matrix
ELISA: Enzyme-linked immunosorbent assay
H&E: Hematoxylin and Eosin
IOD: Integral optical density
OD: Optical density
OVA: Ovalbumin
PAS: Periodic Acid-Schiff
PBS: Phosphate buffered saline
SDF-1: Stromal cell-derived factor 1
VEGF: Vascular endothelial growth factor
WB: Western blotting

## References

[1] Bateman ED, Hurd SS, Barnes PJ, Bousquet J, Drazen JM, FitzGerald JM, et al. Global strategy for asthma management and prevention: GINA executive summary. Eur Respir J. 2008;31(1):143–78. doi: 10.1183/09031936.00138707 . 18166595

[2] Papi A, Brightling C, Pedersen SE, Reddel HK. Asthma. Lancet. 2018;391(10122):783–800. doi: 10.1016/S0140-6736(17)33311-1 . 29273246

[3] Nunes C, Pereira AM, Morais-Almeida M. Asthma costs and social impact. Asthma Res Pract. 2017;3:1. doi: 10.1186/s40733-016-0029-3 . 28078100 PMC5219738

[4] Boonpiyathad T, Sözener ZC, Satitsuksanoa P, Akdis CA. Immunologic mechanisms in asthma. Semin Immunol. 2019;46:101333. doi: 10.1016/j.smim.2019.101333 . 31703832

[5] Guida G, Riccio AM. Immune induction of airway remodeling. Semin Immunol. 2019;46:101346. doi: 10.1016/j.smim.2019.101346 . 31734128

[6] Fehrenbach H, Wagner C, Wegmann M. Airway remodeling in asthma: what really matters. Cell Tissue Res. 2017;367(3):551–69. doi: 10.1007/s00441-016-2566-8 . 28190087 PMC5320023

[7] Hur GY, Broide DH. Genes and Pathways Regulating Decline in Lung Function and Airway Remodeling in Asthma. Allergy Asthma Immunol Res. 2019;11(5):604–21. doi: 10.4168/aair.2019.11.5.604 . 31332973 PMC6658410

[8] Harkness LM, Ashton AW, Burgess JK. Asthma is not only an airway disease, but also a vascular disease. Pharmacol Ther. 2015;148:17–33. doi: 10.1016/j.pharmthera.2014.11.010 . 25460035

[9] Pałgan K, Bartuzi Z. Angiogenesis in bronchial asthma. Int J Immunopathol Pharmacol. 2015;28(3):415–20. doi: 10.1177/0394632015580907 . 25875602

[10] Eldridge L, Wagner EM. Angiogenesis in the lung. J Physiol. 2019;597(4):1023–32. doi: 10.1113/JP275860 . 30022479 PMC6376070

[11] Janssens R, Struyf S, Proost P. Pathological roles of the homeostatic chemokine CXCL12. Cytokine Growth Factor Rev. 2018;44:51–68. doi: 10.1016/j.cytogfr.2018.10.004 . 30396776

[12] Laddha AP, Kulkarni YA. VEGF and FGF-2: Promising targets for the treatment of respiratory disorders. Respir Med. 2019;156:33–46. doi: 10.1016/j.rmed.2019.08.003 . 31421589

[13] Saadat S, Mohamadian Roshan N, Aslani MR, Boskabady MH. Rosuvastatin suppresses cytokine production and lung inflammation in asthmatic, hyperlipidemic and asthmatic-hyperlipidemic rat models. Cytokine. 2020;128:154993. doi: 10.1016/j.cyto.2020.154993 . 32007867

[14] Mohammadian M, Sadeghipour HR, Jahromi GP, Jafari M, Nejad AK, Khamse S, et al. Simvastatin and bone marrow-derived mesenchymal stem cells (BMSCs) affects serum IgE and lung cytokines levels in sensitized mice. Cytokine. 2019;113:83–8. doi: 10.1016/j.cyto.2018.06.016 29914792

[15] Hashemi M, Hoshyar R, Ande SR, Chen QM, Solomon C, Zuse A, et al. Mevalonate Cascade and its Regulation in Cholesterol Metabolism in Different Tissues in Health and Disease. Curr Mol Pharmacol. 2017;10(1):13–26. doi: 10.2174/1874467209666160112123746 . 26758949

[16] Chruściel P, Sahebkar A, Rembek-Wieliczko M, Serban MC, Ursoniu S, Mikhailidis DP, et al. Impact of statin therapy on plasma adiponectin concentrations: A systematic review and meta-analysis of 43 randomized controlled trial arms. Atherosclerosis. 2016;253:194–208. doi: 10.1016/j.atherosclerosis.2016.07.897 . 27498397

[17] Parizadeh SM, Azarpazhooh MR, Moohebati M, Nematy M, Ghayour-Mobarhan M, Tavallaie S, et al. Simvastatin therapy reduces prooxidant-antioxidant balance: results of a placebo-controlled cross-over trial. Lipids. 2011;46(4):333–40. doi: 10.1007/s11745-010-3517-x . 21207250

[18] Wang JY, Yao TC, Tsai YT, Wu AC, Tsai HJ. Increased Dose and Duration of Statin Use Is Associated with Decreased Asthma-Related Emergency Department Visits and Hospitalizations. J Allergy Clin Immunol Pract. 2018;6(5):1588–95 e1. doi: 10.1016/j.jaip.2017.12.017 . 29426752 PMC7032037

[19] Kim JH, Wee JH, Choi HG, Park JY, Hwang YI, Jang SH, et al. Association Between Statin Medication and Asthma/Asthma Exacerbation in a National Health Screening Cohort. J Allergy Clin Immunol Pract. 2021;9(7):2783–91. doi: 10.1016/j.jaip.2021.04.014 . 33894391

[20] Zhang QX, Zhang HF, Lu XT, Zhao J, Xu QX. Statins improve asthma symptoms by suppressing inflammation: a meta-analysis based on RCTs. Eur Rev Med Pharmacol Sci. 2022;26(22):8401–10. doi: 10.26355/eurrev_202211_30376 . 36459023

[21] Maneechotesuwan K, Kasetsinsombat K, Wongkajornsilp A, Barnes PJ. Role of autophagy in regulating interleukin-10 and the responses to corticosteroids and statins in asthma. Clin Exp Allergy. 2021;51(12):1553–65. doi: 10.1111/cea.13825 . 33423318

[22] Lee HY, Lee EG, Hur J, Rhee CK, Kim YK, Lee SY, et al. Pravastatin alleviates allergic airway inflammation in obesity-related asthma mouse model. Exp Lung Res. 2019;45(9–10):275–87. doi: 10.1080/01902148.2019.1675807 . 31608695

[23] Li HX, Liang XY, Wu JH, Yuan YP, Gao Y, Cai SH. Simvastatin attenuates acute lung injury by activation of A2B adenosine receptor. Toxicol Appl Pharmacol. 2021;422:115460. doi: 10.1016/j.taap.2021.115460 . 33774062

[24] Jha A, Ryu MH, Oo O, Bews HJ, Carlson JC, Schwartz J, et al. Prophylactic benefits of systemically delivered simvastatin treatment in a house dust mite challenged murine model of allergic asthma. Br J Pharmacol. 2018;175(7):1004–16. doi: 10.1111/bph.14140 . 29318574 PMC5843706

[25] Cheng AW, Tan X, Sun JY, Gu CM, Liu C, Guo X. Catechin attenuates TNF-α induced inflammatory response via AMPK-SIRT1 pathway in 3T3-L1 adipocytes. PLoS One. 2019;14(5):e0217090. doi: 10.1371/journal.pone.0217090 . 31100089 PMC6524818

[26] Bobba A, Casalino E, Amadoro G, Petragallo VA, Atlante A. AMPK is activated early in cerebellar granule cells undergoing apoptosis and influences VADC1 phosphorylation status and activity. Apoptosis. 2017;22(9):1069–78. doi: 10.1007/s10495-017-1389-8 . 28643197

[27] Gwinn DM, Shackelford DB, Egan DF, Mihaylova MM, Mery A, Vasquez DS, et al. AMPK phosphorylation of raptor mediates a metabolic checkpoint. Mol Cell. 2008;30(2):214–26. doi: 10.1016/j.molcel.2008.03.003 . 18439900 PMC2674027

[28] Hu R, Wang MQ, Ni SH, Wang M, Liu LY, You HY, et al. Salidroside ameliorates endothelial inflammation and oxidative stress by regulating the AMPK/NF-κB/NLRP3 signaling pathway in AGEs-induced HUVECs. Eur J Pharmacol. 2020;867:172797. doi: 10.1016/j.ejphar.2019.172797 . 31747547

[29] Sanz-Ezquerro JJ, Cuenda A. p38 Signalling Pathway. Int J Mol Sci. 2021;22(3). doi: 10.3390/ijms22031003 . 33498296 PMC7863928

[30] Dong L, Wang Y, Zheng T, Pu Y, Ma Y, Qi X, et al. Hypoxic hUCMSC-derived extracellular vesicles attenuate allergic airway inflammation and airway remodeling in chronic asthma mice. Stem Cell Res Ther. 2021;12(1):4. doi: 10.1186/s13287-020-02072-0 . 33407872 PMC7789736

[31] Zhu T, Zhang W, Wang DX, Huang NW, Bo H, Deng W, et al. Rosuvastatin attenuates mucus secretion in a murine model of chronic asthma by inhibiting the gamma-aminobutyric acid type A receptor. Chin Med J (Engl). 2012;125(8):1457–64. . 22613653

[32] Dugger KJ, Chrisman T, Sayner SL, Chastain P, Watson K, Estes R. Beta-2 adrenergic receptors increase TREG cell suppression in an OVA-induced allergic asthma mouse model when mice are moderate aerobically exercised. BMC Immunol. 2018;19(1):9. doi: 10.1186/s12865-018-0244-1 . 29452585 PMC5816563

[33] Ramos-Ramírez P, Noreby M, Liu J, Ji J, Abdillahi SM, Olsson H, et al. A new house dust mite-driven and mast cell-activated model of asthma in the guinea pig. Clin Exp Allergy. 2020;50(10):1184–95. doi: 10.1111/cea.13713 . 32691918

[34] Li N, He Y, Yang G, Yu Q, Li M. Role of TRPC1 channels in pressure-mediated activation of airway remodeling. Respir Res. 2019;20(1):91. doi: 10.1186/s12931-019-1050-x . 31092255 PMC6518742

[35] Ma W, Jin Q, Guo H, Han X, Xu L, Lu S, et al. Metformin Ameliorates Inflammation and Airway Remodeling of Experimental Allergic Asthma in Mice by Restoring AMPKalpha Activity. Front Pharmacol. 2022;13:780148. doi: 10.3389/fphar.2022.780148 . 35153777 PMC8830934

[36] Huang FY, Dai SZ, Xu WT, Xiong W, Sun Y, Huang YH, et al. 3’-epi-12beta-hydroxyfroside-mediated autophagy degradation of RIPK1/RIPK3 necrosomes leads to anergy of immunogenic cell death in triple-negative breast cancer cells. Pharmacol Res. 2023;187:106613. doi: 10.1016/j.phrs.2022.106613 . 36535569

[37] Wang JY, Chen H, Dai SZ, Huang FY, Lin YY, Wang CC, et al. Immunotherapy combining tumor and endothelium cell lysis with immune enforcement by recombinant MIP-3alpha Newcastle disease virus in a vessel-targeting liposome enhances antitumor immunity. J Immunother Cancer. 2022;10(3):e003950. doi: 10.1136/jitc-2021-003950 . 35256516 PMC8905871

[38] Toussaint M, Jackson DJ, Swieboda D, Guedan A, Tsourouktsoglou TD, Ching YM, et al. Host DNA released by NETosis promotes rhinovirus-induced type-2 allergic asthma exacerbation. Nat Med. 2017;23(6):681–91. doi: 10.1038/nm.4332 . 28459437 PMC5821220

[39] Xu L, Dong XW, Shen LL, Li FF, Jiang JX, Cao R, et al. Simvastatin delivery via inhalation attenuates airway inflammation in a murine model of asthma. Int Immunopharmacol. 2012;12(4):556–64. doi: 10.1016/j.intimp.2012.01.012 . 22326624

[40] Zeki AA, Bratt JM, Rabowsky M, Last JA, Kenyon NJ. Simvastatin inhibits goblet cell hyperplasia and lung arginase in a mouse model of allergic asthma: a novel treatment for airway remodeling? Transl Res. 2010;156(6):335–49. doi: 10.1016/j.trsl.2010.09.003 . 21078495 PMC2990975

[41] Gu W, Cui R, Ding T, Li X, Peng J, Xu W, et al. Simvastatin alleviates airway inflammation and remodelling through up-regulation of autophagy in mouse models of asthma. Respirology. 2017;22(3):533–41. doi: 10.1111/resp.12926 . 27782356

[42] Zhu B, Ma AQ, Yang L, Dang XM. Atorvastatin attenuates bleomycin-induced pulmonary fibrosis via suppressing iNOS expression and the CTGF (CCN2)/ERK signaling pathway. Int J Mol Sci. 2013;14(12):24476–91. doi: 10.3390/ijms141224476 . 24351828 PMC3876122

[43] Hamblin MJ, Eberlein M, Black K, Hallowell R, Collins S, Chan-Li Y, et al. Lovastatin Inhibits Low Molecular Weight Hyaluronan Induced Chemokine Expression via LFA-1 and Decreases Bleomycin-Induced Pulmonary Fibrosis. Int J Biomed Sci. 2014;10(3):146–57. doi: 10.1007/s00281-009-0150-y . 25324695 PMC4199473

[44] Karamanos NK, Theocharis AD, Piperigkou Z, Manou D, Passi A, Skandalis SS, et al. A guide to the composition and functions of the extracellular matrix. Febs j. 2021;288(24):6850–912. doi: 10.1111/febs.15776 . 33605520

[45] Dolkart O, Amar E, Shapira S, Marmor S, Steinberg EL, Weinbroum AA. Protective effects of rosuvastatin in a rat model of lung contusion: Stimulation of the cyclooxygenase 2-prostaglandin E-2 pathway. Surgery. 2015;157(5):944–53. doi: 10.1016/j.surg.2014.12.017 . 25724093

[46] Ekerbicer N, Gurpinar T, Sisman AR, Guvendi G, Camsari UM, Uysal N. Statins reduce testicular and ocular VEGF: A potential compromise to microcirculation. Microvasc Res. 2018;119:60–3. doi: 10.1016/j.mvr.2018.04.006 . 29678729

[47] Bellosta S, Ferri N, Bernini F, Paoletti R, Corsini A. Non-lipid-related effects of statins. Ann Med. 2000;32(3):164–76. doi: 10.3109/07853890008998823 . 10821323

[48] Zhu L, Chen X, Chong L, Kong L, Wen S, Zhang H, et al. Adiponectin alleviates exacerbation of airway inflammation and oxidative stress in obesity-related asthma mice partly through AMPK signaling pathway. Int Immunopharmacol. 2019;67:396–407. doi: 10.1016/j.intimp.2018.12.030 . 30584969

[49] Xing J, Wang Q, Coughlan K, Viollet B, Moriasi C, Zou MH. Inhibition of AMP-activated protein kinase accentuates lipopolysaccharide-induced lung endothelial barrier dysfunction and lung injury in vivo. Am J Pathol. 2013;182(3):1021–30. doi: 10.1016/j.ajpath.2012.11.022 . 23306156 PMC3589075

[50] Wang H, Shen X, Tian G, Shi X, Huang W, Wu Y, et al. AMPKα2 deficiency exacerbates long-term PM(2.5) exposure-induced lung injury and cardiac dysfunction. Free Radic Biol Med. 2018;121:202–14. doi: 10.1016/j.freeradbiomed.2018.05.008 . 29753072

[51] Fu H, Alabdullah M, Großmann J, Spieler F, Abdosh R, Lutz V, et al. The differential statin effect on cytokine production of monocytes or macrophages is mediated by differential geranylgeranylation-dependent Rac1 activation. Cell Death Dis. 2019;10(12):880. doi: 10.1038/s41419-019-2109-9 . 31754207 PMC6872739

[52] Bagnato G, Bitto A, Pizzino G, Irrera N, Sangari D, Cinquegrani M, et al. Simvastatin attenuates the development of pulmonary and cutaneous fibrosis in a murine model of systemic sclerosis. Rheumatology (Oxford). 2013;52(8):1377–86. doi: 10.1093/rheumatology/ket144 . 23620550

[53] Jang HJ, Lee DY, Loloci G, Jeong J, Choi WI. Association between the use of statins and risk of interstitial lung disease/idiopathic pulmonary fibrosis: time-dependent analysis of population-based nationwide data. Eur Respir J. 2023;62(1). doi: 10.1183/13993003.00291-2023 . 37202155

[54] Varricchi G, Ferri S, Pepys J, Poto R, Spadaro G, Nappi E, et al. Biologics and airway remodeling in severe asthma. Allergy. 2022;77(12):3538–52. doi: 10.1111/all.15473 . 35950646 PMC10087445

[55] Zahedipour F, Butler AE, Rizzo M, Sahebkar A. Statins and angiogenesis in non-cardiovascular diseases. Drug Discov Today. 2022;27(10):103320. doi: 10.1016/j.drudis.2022.07.005 . 35850434

[56] Liu JN, Suh DH, Yang EM, Lee SI, Park HS, Shin YS. Attenuation of airway inflammation by simvastatin and the implications for asthma treatment: is the jury still out? Exp Mol Med. 2014;46(9):e113. doi: 10.1038/emm.2014.55 . 25213768 PMC4183942

[57] Saheb Sharif-Askari N, Alabed M, Selvakumar B, Mdkhana B, Salam Bayram O, Kalaji Z, et al. Simvastatin reduced infiltration of memory subsets of T lymphocytes in the lung tissue during Th2 allergic inflammation. Int Immunopharmacol. 2022;113(Pt A):109347. doi: 10.1016/j.intimp.2022.109347 . 36332451

