# Supplementary figures and images for: Rosuvastatin attenuates airway inflammation and remodeling in a chronic allergic asthma model through modulation of the AMPKα signaling pathway
Source: PLoS One. 2024 Jun 24;19(6):e0305863. doi: 10.1371/journal.pone.0305863 (PMC11195969; doi:10.1371/journal.pone.0305863)

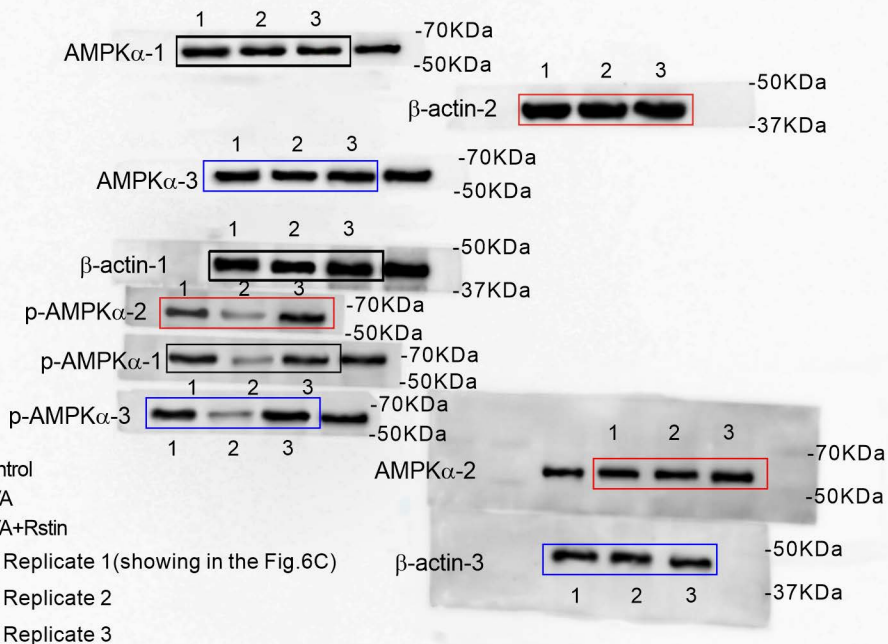

Supplement: S1 Raw images — (PDF) [file pone.0305863.s001.pdf]
