# Supplementary material for: Rosuvastatin attenuates airway inflammation and remodeling in a chronic allergic asthma model through modulation of the AMPKα signaling pathway
Source: PLoS One. 2024 Jun 24;19(6):e0305863. doi: 10.1371/journal.pone.0305863 (PMC11195969; doi:10.1371/journal.pone.0305863)
Supplement: S1 Data — (PDF) [file pone.0305863.s002.pdf]

Rosuvastatin ameliorates airway inflammation and remodeling in ovalbumin-induced chronic allergic mice, involving the AMPKα signaling pathway

Lei Zhang[1¶], Feng-Ying Huang[2¶], Shu-Zhen Dai[2], Lin Wang[1], Xiangdong Zhou[1], Zhen-You Zheng[3], Qi Li[1*], Guang-Hong Tan[2*], Cai-Chun Wang[1*]

[1] Department of Respiratory Medicine, The First Affiliated Hospital of Hainan Medical University & Hainan Province Clinical Medical Center of Respiratory Disease, Haikou, China

[2] NHC Key Laboratory of Tropical Disease Control, School of Tropical Medicine & The Second Affiliated Hospital, Hainan Medical University, Haikou, China.

[3] Department of Ophthalmology, The First Affiliated Hospital of Hainan Medical University, Haikou, China

Source data and the results of statistical analysis……….................1-25

Figure 1C

	Group A	Group B	Group C
	Normal	OVA	OVA+ROS
1	0.343	3.436	1.321
2	0.337	3.295	1.693
3	0.402	3.151	1.404
4	0.367	3.426	1.233
5	0.356	3.660	1.488

ANOVA results | Multiple comparisons

	Ordinary one-way ANOVA<br>ANOVA results				
1	Table Analyzed	Figure 1C			
2	Data sets analyzed	A-C			
3					
4	**ANOVA summary**				
5	F	528.0			
6	P value	<0.0001			
7	P value summary	****			
8	Significant diff. among means (P < 0.05)'	Yes			
9	R squared	0.9888			
10					
11	**Brown-Forsythe test**				
12	F (DFn, DFd)	2.152 (2, 12)			
13	P value	0.1590			
14	P value summary	ns			
15	Are SDs significantly different (P < 0.05)'	No			
16					
17	**Bartlett's test**				
18	Bartlett's statistic (corrected)	9.856			
19	P value	0.0072			
20	P value summary	**			
21	Are SDs significantly different (P < 0.05)'	Yes			
22					
23	**ANOVA table**	**SS**	**DF**	**MS**	**F (DFn, DFd)**	**P value**
24	Treatment (between columns)	23.67	2	11.83	F (2, 12) = 528.0	P<0.0001
25	Residual (within columns)	0.2689	12	0.02241		
26	Total	23.93	14			
27						
28	**Data summary**					
29	Number of treatments (columns)	3				
30	Number of values (total)	15				

ANOVA results | Multiple comparisons

	Ordinary one-way ANOVA<br>Multiple comparisons								
1	Number of families	1							
2	Number of comparisons per family	3							
3	Alpha	0.05							
4									
5	**Tukey's multiple comparisons test**	**Mean Diff.**	**95.00% CI of diff.**	**Below threshold?**	**Summar**	**Adjusted P Value**			
6	Normal vs. OVA	-3.033	-3.285 to -2.780	Yes	****	<0.0001	A-B		
7	Normal vs. OVA+ROS	-1.067	-1.319 to -0.8142	Yes	****	<0.0001	A-C		
8	OVA vs. OVA+ROS	1.966	1.713 to 2.218	Yes	****	<0.0001	B-C		
9									
10	**Test details**	**Mean 1**	**Mean 2**	**Mean Diff.**	**SE of diff**	**n1**	**n2**	**q**	**DF**
11	Normal vs. OVA	0.3610	3.394	-3.033	0.09468	5	5	45.30	12
12	Normal vs. OVA+ROS	0.3610	1.428	-1.067	0.09468	5	5	15.94	12
13	OVA vs. OVA+ROS	3.394	1.428	1.966	0.09468	5	5	29.36	12
14									

Figure 1D

	Group A	Group B	Group C
	Normal	OVA	OVA+ROS
1	13.691	28.756	23.640
2	14.151	35.082	24.616
3	19.536	40.591	23.444
4	15.592	39.229	17.271
5	17.105	41.865	21.651

	Ordinary one-way ANOVA<br>ANOVA results					
10						
11	**Brown-Forsythe test**					
12	F (DFn, DFd)	0.7336 (2, 12)				
13	P value	0.5005				
14	P value summary	ns				
15	Are SDs significantly different (P < 0.05)'	No				
16						
17	**Bartlett's test**					
18	Bartlett's statistic (corrected)	2.637				
19	P value	0.2675				
20	P value summary	ns				
21	Are SDs significantly different (P < 0.05)'	No				
22						
23	**ANOVA table**	**SS**	**DF**	**MS**	**F (DFn, DFd**	**P value**
24	Treatment (between columns)	1178	2	588.8	F (2, 12) = 4	P<0.0001
25	Residual (within columns)	169.8	12	14.15		
26	Total	1347	14			
27						
28	**Data summary**					
29	Number of treatments (columns)	3				
30	Number of values (total)	15				

	Ordinary one-way ANOVA<br>Multiple comparisons								
1	Number of families	1							
2	Number of comparisons per family	3							
3	Alpha	0.05							
4									
5	**Tukey's multiple comparisons test**	**Mean Diff.**	**95.00% CI of diff.**	**Below threshold?**	**Summary**				
6	Normal vs. OVA	-21.09	-27.44 to -14.74	Yes	****		A-B		
7	Normal vs. OVA+ROS	-6.109	-12.46 to 0.2374	No	ns		A-C		
8	OVA vs. OVA+ROS	14.98	8.633 to 21.33	Yes	***		B-C		
9									
10	**Test details**	**Mean 1**	**Mean 2**	**Mean Diff.**	**SE of diff.**	**n1**	**n2**	**q**	**DF**
11	Normal vs. OVA	16.02	37.10	-21.09	2.379	5	5	12.54	12
12	Normal vs. OVA+ROS	16.02	22.12	-6.109	2.379	5	5	3.632	12
13	OVA vs. OVA+ROS	37.10	22.12	14.98	2.379	5	5	8.905	12

Figure 1E

	Group A	Group B	Group C
	Normal	OVA	OVA+ROS
1	2.570	6.652	5.374
2	3.025	9.710	4.946
3	2.592	7.795	4.642
4	2.560	8.986	4.847
5	2.298	9.528	4.976

Ordinary one-way ANOVA
ANOVA results

1	Table Analyzed	Figure 1E				
2	Data sets analyzed	A-C				
3						
4	**ANOVA summary**					
5	F	73.95				
6	P value	<0.0001				
7	P value summary	****				
8	Significant diff. among means (P < 0.05)'	Yes				
9	R squared	0.9250				
10						
11	**Brown-Forsythe test**					
12	F (DFn, DFd)	3.733 (2, 12)				
13	P value	0.0549				
14	P value summary	ns				
15	Are SDs significantly different (P < 0.05)'	No				
16						
17	**Bartlett's test**					
18	Bartlett's statistic (corrected)	11.83				
19	P value	0.0027				
20	P value summary	**				
21	Are SDs significantly different (P < 0.05)'	Yes				
22						
23	**ANOVA table**	**SS**	**DF**	**MS**	**F (DFn, DFd)**	**P value**
24	Treatment (between columns)	89.03	2	44.51	F (2, 12) = 73.95	P<0.0001
25	Residual (within columns)	7.223	12	0.6019		
26	Total	96.25	14			
27						
28	**Data summary**					
29	Number of treatments (columns)	3				
30	Number of values (total)	15				

Ordinary one-way ANOVA
Multiple comparisons

4									
5	**Tukey's multiple comparisons t**	**Mean Diff.**	**95.00% CI of diff.**	**Below threshold?**	**Summary**	**Adjusted P Value**			
6	Normal vs. OVA	-5.925	-7.234 to -4.616	Yes	****	<0.0001	A-B		
7	Normal vs. OVA+ROS	-2.348	-3.657 to -1.039	Yes	**	0.0012	A-C		
8	OVA vs. OVA+ROS	3.577	2.268 to 4.886	Yes	****	<0.0001	B-C		
9									
10	**Test details**	**Mean 1**	**Mean 2**	**Mean Diff.**	**SE of diff**	**n1**	**n2**	**q**	**DF**
11	Normal vs. OVA	2.609	8.534	-5.925	0.4907	5	5	17.08	12
12	Normal vs. OVA+ROS	2.609	4.957	-2.348	0.4907	5	5	6.767	12
13	OVA vs. OVA+ROS	8.534	4.957	3.577	0.4907	5	5	10.31	12

Figure 2B

	Group A	Group B	Group C
	Normal	OVA	OVA+ROS
1	0.156	3.248	1.314
2	0.162	3.251	1.373
3	0.183	2.714	0.903
4	0.264	3.402	1.467
5	0.162	3.189	1.279

ANOVAresults | Multiple comparisons | Descriptive statistics

	Ordinary one-way ANOVA<br>ANOVA results					
1	Table Analyzed	Figure 2B				
2	Data sets analyzed	A-C				
3						
4	**ANOVA summary**					
5	F	290.4				
6	P value	<0.0001				
7	P value summary	****				
8	Significant diff. among means (P < 0.05)'	Yes				
9	R squared	0.9798				
10						
11	**Brown-Forsythe test**					
12	F (DFn, DFd)	0.8481 (2, 12)				
13	P value	0.4524				
14	P value summary	ns				
15	Are SDs significantly different (P < 0.05)'	No				
16						
17	**Bartlett's test**					
18	Bartlett's statistic (corrected)	7.976				
19	P value	0.0185				
20	P value summary	*				
21	Are SDs significantly different (P < 0.05)'	Yes				
22						
23	**ANOVA table**	**SS**	**DF**	**MS**	**F (DFn, DFd)**	**P value**
24	Treatment (between columns)	22.68	2	11.34	F (2, 12) = 290.4	P<0.0001
25	Residual (within columns)	0.4686	12	0.03905		
26	Total	23.15	14			
27						
28	**Data summary**					
29	Number of treatments (columns)	3				
30	Number of values (total)	15				

	Ordinary one-way ANOVA<br>Multiple comparisons								
1	Number of families	1							
2	Number of comparisons per family	3							
3	Alpha	0.05							
4									
5	**Tukey's multiple comparisons test**	**Mean Diff.**	**95.00% CI of diff.**	**Below threshold?**	**Summary**	**Adjusted P Value**			
6	Normal vs. OVA	-2.975	-3.309 to -2.642	Yes	****	<0.0001	A-B		
7	Normal vs. OVA+ROS	-1.082	-1.415 to -0.7484	Yes	****	<0.0001	A-C		
8	OVA vs. OVA+ROS	1.894	1.560 to 2.227	Yes	****	<0.0001	B-C		
9									
10	**Test details**	**Mean 1**	**Mean 2**	**Mean Diff.**	**SE of diff**	**n1**	**n2**	**q**	**DF**
11	Normal vs. OVA	0.1854	3.161	-2.975	0.1250	5	5	33.67	12
12	Normal vs. OVA+ROS	0.1854	1.267	-1.082	0.1250	5	5	12.24	12
13	OVA vs. OVA+ROS	3.161	1.267	1.894	0.1250	5	5	21.43	12

Figure 2C

	Group A	Group B	Group C
	Normal	OVA	OVA+ROS
1	0.875	16.522	12.196
2	1.519	19.233	13.164
3	1.258	20.034	14.980
4	1.316	19.975	10.533
5	1.336	22.536	10.449

	Ordinary one-way ANOVA<br>ANOVA results					
1	Table Analyzed	Figure 2C				
2	Data sets analyzed	A-C				
3						
4	**ANOVA summary**					
5	F	154.7				
6	P value	<0.0001				
7	P value summary	****				
8	Significant diff. among means (P < 0.05)'	Yes				
9	R squared	0.9627				
10						
11	**Brown-Forsythe test**					
12	F (DFn, DFd)	2.229 (2, 12)				
13	P value	0.1503				
14	P value summary	ns				
15	Are SDs significantly different (P < 0.05)'	No				
16						
17	**Bartlett's test**					
18	Bartlett's statistic (corrected)	11.23				
19	P value	0.0037				
20	P value summary	**				
21	Are SDs significantly different (P < 0.05)'	Yes				
22						
23	**ANOVA table**	**SS**	**DF**	**MS**	**F (DFn, DFd)**	**P value**
24	Treatment (between columns)	857.2	2	428.6	F (2, 12) = 154.7	P<0.0001
25	Residual (within columns)	33.25	12	2.770		
26	Total	890.4	14			
27						
28	**Data summary**					
29	Number of treatments (columns)	3				
30	Number of values (total)	15				

	Ordinary one-way ANOVA<br>Multiple comparisons									
1	Number of families	1								
2	Number of comparisons per family	3								
3	Alpha	0.05								
4										
5	**Tukey's multiple comparisons test**	**Mean Diff**	**95.00% CI of diff.**	**Below threshold?**	**Summary**	**Adjusted P Value**				
6	Normal vs. OVA	-18.40	-21.21 to -15.59	Yes	****	<0.0001	A-B			
7	Normal vs. OVA+ROS	-11.00	-13.81 to -8.195	Yes	****	<0.0001	A-C			
8	OVA vs. OVA+ROS	7.396	4.587 to 10.20	Yes	****	<0.0001	B-C			
9										
10	**Test details**	**Mean 1**	**Mean 2**	**Mean Diff.**	**SE of diff.**	**n1**	**n2**	**q**	**DF**	
11	Normal vs. OVA	1.261	19.66	-18.40	1.053	5	5	24.72	12	
12	Normal vs. OVA+ROS	1.261	12.26	-11.00	1.053	5	5	14.78	12	
13	OVA vs. OVA+ROS	19.66	12.26	7.396	1.053	5	5	9.935	12	

Figure 3B

	Group A	Group B	Group C
	Normal	OVA	OVA+ROS
1	0.023	0.082	0.043
2	0.020	0.083	0.034
3	0.022	0.069	0.049
4	0.014	0.069	0.040
5	0.018	0.067	0.044

	Ordinary one-way ANOVA<br>ANOVA results					
1	Table Analyzed	Figure 3B				
2	Data sets analyzed	A-C				
3						
4	**ANOVA summary**					
5	F	108.2				
6	P value	<0.0001				
7	P value summary	****				
8	Significant diff. among means (P < 0.05)'	Yes				
9	R squared	0.9475				
10						
11	**Brown-Forsythe test**					
12	F (DFn, DFd)	0.5706 (2, 12)				
13	P value	0.5798				
14	P value summary	ns				
15	Are SDs significantly different (P < 0.05)'	No				
16						
17	**Bartlett's test**					
18	Bartlett's statistic (corrected)	2.045				
19	P value	0.3598				
20	P value summary	ns				
21	Are SDs significantly different (P < 0.05)'	No				
22						
23	**ANOVA table**	**SS**	**DF**	**MS**	**F (DFn, DFd)**	**P value**
24	Treatment (between columns)	0.007527	2	0.003763	F (2, 12) = 108.2	P<0.0001
25	Residual (within columns)	0.0004172	12	3.477e-005		
26	Total	0.007944	14			
27						
28	**Data summary**					
29	Number of treatments (columns)	3				
30	Number of values (total)	15				

	Ordinary one-way ANOVA<br>Multiple comparisons								
1	Number of families	1							
2	Number of comparisons per family	3							
3	Alpha	0.05							
4									
5	**Tukey's multiple comparisons test**	**Mean Diff.**	**95.00% CI of diff.**	**Below threshold?**	**Summary**	**Adjusted P Value**			
6	Normal vs. OVA	-0.05460	-0.06455 to -0.04465	Yes	****	<0.0001	A-B		
7	Normal vs. OVA+ROS	-0.02260	-0.03255 to -0.01265	Yes	***	0.0002	A-C		
8	OVA vs. OVA+ROS	0.03200	0.02205 to 0.04195	Yes	****	<0.0001	B-C		
9									
10	**Test details**	**Mean 1**	**Mean 2**	**Mean Diff.**	**SE of diff.**	**n1**	**n2**	**q**	**DF**
11	Normal vs. OVA	0.01940	0.07400	-0.05460	0.003729	5	5	20.71	12
12	Normal vs. OVA+ROS	0.01940	0.04200	-0.02260	0.003729	5	5	8.571	12
13	OVA vs. OVA+ROS	0.07400	0.04200	0.03200	0.003729	5	5	12.14	12

Figure 3C

	Group A	Group B	Group C
	Normal	OVA	OVA+ROS
1	0.00918	0.02492	0.01981
2	0.00747	0.02807	0.01163
3	0.00660	0.02055	0.01639
4	0.00884	0.02590	0.01954
5	0.00856	0.02601	0.01871

Ordinary one-way ANOVA
ANOVA results

4	**ANOVA summary**					
5	F	52.81				
6	P value	<0.0001				
7	P value summary	****				
8	Significant diff. among means (P < 0.05)'	Yes				
9	R squared	0.8980				
10						
11	**Brown-Forsythe test**					
12	F (DFn, DFd)	0.6239 (2, 12)				
13	P value	0.5524				
14	P value summary	ns				
15	Are SDs significantly different (P < 0.05)'	No				
16						
17	**Bartlett's test**					
18	Bartlett's statistic (corrected)	4.070				
19	P value	0.1307				
20	P value summary	ns				
21	Are SDs significantly different (P < 0.05)'	No				
22						
23	**ANOVA table**	**SS**	**DF**	**MS**	**F (DFn, DFd**	**P value**
24	Treatment (between columns)	0.0007203	2	0.0003602	F (2, 12) = 5.	P<0.0001
25	Residual (within columns)	8.184e-005	12	6.820e-006		
26	Total	0.0008022	14			
27						
28	**Data summary**					
29	Number of treatments (columns)	3				
30	Number of values (total)	15				

Ordinary one-way ANOVA
Multiple comparisons

4									
5	**Tukey's multiple comparisons t**	**Mean Diff.**	**95.00% CI of diff.**	**Below threshold?**	**Summary**	**Adjusted P Value**			
6	Normal vs. OVA	-0.01696	-0.02137 to -0.01255	Yes	****	<0.0001	A-B		
7	Normal vs. OVA+ROS	-0.009086	-0.01349 to -0.00468(	Yes	***	0.0004	A-C		
8	OVA vs. OVA+ROS	0.007874	0.003468 to 0.01228	Yes	**	0.0012	B-C		
9									
10	**Test details**	**Mean 1**	**Mean 2**	**Mean Diff.**	**SE of diff.**	**n1**	**n2**	**q**	**DF**
11	Normal vs. OVA	0.008130	0.02509	-0.01696	0.001652	5	5	14.52	12
12	Normal vs. OVA+ROS	0.008130	0.01722	-0.009086	0.001652	5	5	7.780	12
13	OVA vs. OVA+ROS	0.02509	0.01722	0.007874	0.001652	5	5	6.742	12

Figure 3D

	Group A	Group B	Group C
	Normal	OVA	OVA+ROS
1	0.010	0.043	0.022
2	0.013	0.051	0.029
3	0.007	0.030	0.030
4	0.010	0.052	0.026
5	0.010	0.041	0.027

	Ordinary one-way ANOVA<br>ANOVA results					
1	Table Analyzed	Figure 3D				
2	Data sets analyzed	A-C				
3						
4	**ANOVA summary**					
5	F	44.74				
6	P value	<0.0001				
7	P value summary	****				
8	Significant diff. among means (P < 0.05)?	Yes				
9	R squared	0.8818				
10						
11	**Brown-Forsythe test**					
12	F (DFn, DFd)	3.291 (2, 12)				
13	P value	0.0725				
14	P value summary	ns				
15	Are SDs significantly different (P < 0.05)?	No				
16						
17	**Bartlett's test**					
18	Bartlett's statistic (corrected)	7.807				
19	P value	0.0202				
20	P value summary	*				
21	Are SDs significantly different (P < 0.05)?	Yes				
22						
23	**ANOVA table**	**SS**	**DF**	**MS**	**F (DFn, DFd)**	**P value**
24	Treatment (between columns)	0.002789	2	0.001394	F (2, 12) = 44.74	P<0.0001
25	Residual (within columns)	0.0003740	12	3.117e-005		
26	Total	0.003163	14			
27						
28	**Data summary**					
29	Number of treatments (columns)	3				
30	Number of values (total)	15				

	Ordinary one-way ANOVA<br>Multiple comparisons								
1	Number of families	1							
2	Number of comparisons per family	3							
3	Alpha	0.05							
4									
5	**Tukey's multiple comparisons test**	**Mean Diff.**	**95.00% CI of diff.**	**Below threshold?**	**Summary**	**Adjusted P Value**			
6	Normal vs. OVA	-0.03340	-0.04282 to -0.02398	Yes	****	<0.0001	A-B		
7	Normal vs. OVA+ROS	-0.01680	-0.02622 to -0.007380	Yes	**	0.0012	A-C		
8	OVA vs. OVA+ROS	0.01660	0.007180 to 0.02602	Yes	**	0.0014	B-C		
9									
10	**Test details**	**Mean 1**	**Mean 2**	**Mean Diff.**	**SE of diff.**	**n1**	**n2**	**q**	**DF**
11	Normal vs. OVA	0.01000	0.04340	-0.03340	0.003531	5	5	13.38	12
12	Normal vs. OVA+ROS	0.01000	0.02680	-0.01680	0.003531	5	5	6.729	12
13	OVA vs. OVA+ROS	0.04340	0.02680	0.01660	0.003531	5	5	6.649	12

Figure 4A

	Group A	Group B	Group C
	Normal	OVA	OVA+ROS
1	25.297	101.868	67.896
2	21.347	81.752	56.484
3	22.035	98.447	68.931
4	23.663	104.677	64.437
5	20.506	99.492	65.964

	Ordinary one-way ANOVA<br>ANOVA results					
1	Table Analyzed	Figure 4A				
2	Data sets analyzed	A-C				
3						
4	**ANOVA summary**					
5	F	193.3				
6	P value	<0.0001				
7	P value summary	****				
8	Significant diff. among means (P < 0.05)'	Yes				
9	R squared	0.9699				
10						
11	**Brown-Forsythe test**					
12	F (DFn, DFd)	0.8256 (2, 12)				
13	P value	0.4614				
14	P value summary	ns				
15	Are SDs significantly different (P < 0.05)?	No				
16						
17	**Bartlett's test**					
18	Bartlett's statistic (corrected)	6.801				
19	P value	0.0334				
20	P value summary	*				
21	Are SDs significantly different (P < 0.05)?	Yes				
22						
23	**ANOVA table**	**SS**	**DF**	**MS**	**F (DFn, DFd)**	**P value**
24	Treatment (between columns)	14020	2	7010	F (2, 12) = 193.3	P<0.0001
25	Residual (within columns)	435.1	12	36.26		
26	Total	14455	14			
27						
28	**Data summary**					
29	Number of treatments (columns)	3				
30	Number of values (total)	15				

	Ordinary one-way ANOVA<br>Multiple comparisons								
1	Number of families	1							
2	Number of comparisons per family	3							
3	Alpha	0.05							
4									
5	**Tukey's multiple comparisons test**	**Mean Diff.**	**95.00% CI of diff.**	**Below threshold?**	**Summary**	**Adjusted P Value**			
6	Normal vs. OVA	-74.68	-84.84 to -64.52	Yes	****	<0.0001	A-B		
7	Normal vs. OVA+ROS	-42.17	-52.33 to -32.01	Yes	****	<0.0001	A-C		
8	OVA vs. OVA+ROS	32.50	22.34 to 42.66	Yes	****	<0.0001	B-C		
9									
10	**Test details**	**Mean 1**	**Mean 2**	**Mean Diff.**	**SE of diff.**	**n1**	**n2**	**q**	**DF**
11	Normal vs. OVA	22.57	97.25	-74.68	3.808	5	5	27.73	12
12	Normal vs. OVA+ROS	22.57	64.74	-42.17	3.808	5	5	15.66	12
13	OVA vs. OVA+ROS	97.25	64.74	32.50	3.808	5	5	12.07	12

Figure 4B

Table format: Grouped	Group A					Group B					Group C				
	Normal					OVA					OVA+ROS				
	A:1	A:2	A:3	A:4	A:5	B:1	B:2	B:3	B:4	B:5	C:1	C:2	C:3	C:4	C:5
1 Macrophage	51.086	49.656	53.375	56.745	57.217	44.098	54.251	47.290	52.886	45.907	50.343	43.327	37.497	48.787	40.207
2 Eosinophil	1.762	2.066	1.805	2.020	2.082	15.631	12.041	15.609	15.012	11.839	8.713	4.922	7.843	7.119	6.823
3 Neutrophil	0.922	0.909	1.150	0.841	1.142	7.534	5.898	5.135	7.372	8.142	2.807	3.197	3.706	3.508	3.137
4 Lymphocyte	14.987	12.139	16.988	12.908	15.889	7.315	6.615	7.748	8.225	7.024	10.320	11.960	13.575	9.572	8.308

	2way ANOVA<br>ANOVA results					
1	Table Analyzed	Figure 4B				
2						
3	**Two-way RM ANOVA**	Matching: Both factors				
4	Assume sphericity?	No				
5	Alpha	0.05				
6						
7	**Source of Variation**	**% of total variation**	**P value**	**P value summary**	**Significant?**	**Geisser-Greenhouse's epsilon**
8	Row Factor	94.61	<0.0001	****	Yes	0.3805
9	Column Factor	0.4310	0.0244	*	Yes	0.8142
10	Row Factor x Column Factor	3.475	0.0009	***	Yes	0.3455
11	Subject x Row Factor	0.4042				
12	Subject x Column Factor	0.2351				
13	Subject	0.08619				
14						
15	**ANOVA table**	**SS**	**DF**	**MS**	**F (DFn, DFd)**	**P value**
16	Row Factor	19689	3	6563	F (1.141, 4.566) = 936.2	P<0.0001
17	Column Factor	89.70	2	44.85	F (1.628, 6.514) = 7.335	P=0.0244
18	Row Factor x Column Factor	723.2	6	120.5	F (2.073, 8.293) = 18.31	P=0.0009
19	Subject x Row Factor	84.12	12	7.010		
20	Subject x Column Factor	48.92	8	6.115		
21	Subject	17.94	4	4.484		
22	Residual	158.0	24	6.582		
23						
24	**Data summary**					
25	Number of columns (Column Factor	3				
26	Number of rows (Row Factor)	4				
27	Number of subjects (Subject)	5				
28	Number of missing values	0				

	2way ANOVA<br>Multiple comparisons									
1	Within each row, compare columns (simple effects within row									
2										
3	Number of families	4								
4	Number of comparisons per family	3								
5	Alpha	0.05								
6										
7	**Tukey's multiple comparisons test**	**Mean Diff.**	**95.00% CI of diff.**	**Below threshold?**	**Summary**	**Adjusted P Value**				
8										
9	Macrophage									
10	Normal vs. OVA	4.729	-4.631 to 14.09	No	ns	0.2802				
11	Normal vs. OVA+ROS	9.584	-1.290 to 20.46	No	ns	0.0731				
12	OVA vs. OVA+ROS	4.854	-6.006 to 15.71	No	ns	0.3475				
13										
14	Eosinophil									
15	Normal vs. OVA	-12.08	-15.35 to -8.810	Yes	***	0.0004				
16	Normal vs. OVA+ROS	-5.137	-7.589 to -2.685	Yes	**	0.0038				
17	OVA vs. OVA+ROS	6.942	5.103 to 8.781	Yes	***	0.0004				
18										
19	Neutrophil									
20	Normal vs. OVA	-5.823	-7.868 to -3.779	Yes	**	0.0012				
21	Normal vs. OVA+ROS	-2.278	-2.821 to -1.736	Yes	***	0.0003				
22	OVA vs. OVA+ROS	3.545	1.178 to 5.912	Yes	*	0.0130				
23										
24	Lymphocyte									
25	Normal vs. OVA	7.197	3.979 to 10.41	Yes	**	0.0030				
26	Normal vs. OVA+ROS	3.835	-0.4206 to 8.091	No	ns	0.0685				
27	OVA vs. OVA+ROS	-3.362	-6.791 to 0.06746	No	ns	0.0532				
28										
29										
30	**Test details**	**Mean 1**	**Mean 2**	**Mean Diff.**	**SE of diff.**	**N1**	**N2**	**q**	**DF**	
31										
32	Macrophage									
33	Normal vs. OVA	53.62	48.89	4.729	2.626	5	5	2.547	4.000	
34	Normal vs. OVA+ROS	53.62	44.03	9.584	3.051	5	5	4.442	4.000	
35	OVA vs. OVA+ROS	48.89	44.03	4.854	3.047	5	5	2.253	4.000	
36										
37	Eosinophil									
38	Normal vs. OVA	1.947	14.03	-12.08	0.9174	5	5	18.62	4.000	
39	Normal vs. OVA+ROS	1.947	7.084	-5.137	0.6879	5	5	10.56	4.000	
40	OVA vs. OVA+ROS	14.03	7.084	6.942	0.5160	5	5	19.03	4.000	

Figure 4C

	Group A	Group B	Group C
	Normal	OVA	OVA+ROS
1	28.764	81.848	49.174
2	34.158	77.580	42.297
3	26.666	83.631	45.741
4	21.632	71.415	41.172
5	33.670	67.329	47.393

	Ordinary one-way ANOVA<br>ANOVA results					
1	Table Analyzed	Figure 4C				
2	Data sets analyzed	A-C				
3						
4	**ANOVA summary**					
5	F	101.1				
6	P value	<0.0001				
7	P value summary	****				
8	Significant diff. among means (P < 0.05)'	Yes				
9	R squared	0.9440				
10						
11	**Brown-Forsythe test**					
12	F (DFn, DFd)	1.116 (2, 12)				
13	P value	0.3592				
14	P value summary	ns				
15	Are SDs significantly different (P < 0.05)'	No				
16						
17	**Bartlett's test**					
18	Bartlett's statistic (corrected)	1.713				
19	P value	0.4246				
20	P value summary	ns				
21	Are SDs significantly different (P < 0.05)'	No				
22						
23	**ANOVA table**	**SS**	**DF**	**MS**	**F (DFn, DFd)**	**P value**
24	Treatment (between columns)	5801	2	2900	F (2, 12) = 101.1	P<0.0001
25	Residual (within columns)	344.2	12	28.69		
26	Total	6145	14			
27						
28	**Data summary**					
29	Number of treatments (columns)	3				
30	Number of values (total)	15				

	Ordinary one-way ANOVA<br>Multiple comparisons								
1	Number of families	1							
2	Number of comparisons per family	3							
3	Alpha	0.05							
4									
5	**Tukey's multiple comparisons test**	**Mean Diff.**	**95.00% CI of diff.**	**Below threshold?**	**Summary**	**Adjusted P Value**			
6	Normal vs. OVA	-47.38	-56.42 to -38.35	Yes	****	<0.0001	A-B		
7	Normal vs. OVA+ROS	-16.18	-25.21 to -7.140	Yes	**	0.0012	A-C		
8	OVA vs. OVA+ROS	31.21	22.17 to 40.24	Yes	****	<0.0001	B-C		
9									
10	**Test details**	**Mean 1**	**Mean 2**	**Mean Diff.**	**SE of diff.**	**n1**	**n2**	**q**	**DF**
11	Normal vs. OVA	28.98	76.36	-47.38	3.387	5	5	19.78	12
12	Normal vs. OVA+ROS	28.98	45.16	-16.18	3.387	5	5	6.754	12
13	OVA vs. OVA+ROS	76.36	45.16	31.21	3.387	5	5	13.03	12

Figure 4D

	Group A	Group B	Group C
	Normal	OVA	OVA+ROS
1	3.899	8.903	6.580
2	4.110	11.119	5.104
3	4.219	8.524	7.325
4	3.890	10.465	6.040
5	3.857	7.582	6.411

Ordinary one-way ANOVA
ANOVA results

1	Table Analyzed	Figure 4D				
2	Data sets analyzed	A-C				
3						
4	**ANOVA summary**					
5	F	38.48				
6	P value	<0.0001				
7	P value summary	****				
8	Significant diff. among means (P < 0.05)'	Yes				
9	R squared	0.8651				
10						
11	**Brown-Forsythe test**					
12	F (DFn, DFd)	3.206 (2, 12)				
13	P value	0.0766				
14	P value summary	ns				
15	Are SDs significantly different (P < 0.05)'	No				
16						
17	**Bartlett's test**					
18	Bartlett's statistic (corrected)	11.20				
19	P value	0.0037				
20	P value summary	**				
21	Are SDs significantly different (P < 0.05)'	Yes				
22						
23	**ANOVA table**	**SS**	**DF**	**MS**	**F (DFn, DFd)**	**P value**
24	Treatment (between columns)	71.30	2	35.65	F (2, 12) = 38.48	P<0.0001
25	Residual (within columns)	11.12	12	0.9264		
26	Total	82.41	14			
27						
28	**Data summary**					
29	Number of treatments (columns)	3				
30	Number of values (total)	15				

Ordinary one-way ANOVA
Multiple comparisons

4										
5	**Tukey's multiple comparisons t**	**Mean Diff.**	**95.00% CI of diff**	**Below threshold?**	**Summary**	**Adjusted P Value**				
6	Normal vs. OVA	-5.324	-6.948 to -3.700	Yes	****	<0.0001	A-B			
7	Normal vs. OVA+ROS	-2.297	-3.921 to -0.6729	Yes	**	0.0069	A-C			
8	OVA vs. OVA+ROS	3.027	1.403 to 4.651	Yes	***	0.0009	B-C			
9										
10	**Test details**	**Mean 1**	**Mean 2**	**Mean Diff.**	**SE of diff.**	**n1**	**n2**	**q**	**DF**	
11	Normal vs. OVA	3.995	9.319	-5.324	0.6087	5	5	12.37	12	
12	Normal vs. OVA+ROS	3.995	6.292	-2.297	0.6087	5	5	5.336	12	
13	OVA vs. OVA+ROS	9.319	6.292	3.027	0.6087	5	5	7.031	12	

Figure 4E

Group A	Group B	Group C
Normal	OVA	OVA+ROS
2.001	11.170	9.390
1.547	13.566	8.377
2.118	11.853	9.281
2.193	15.618	6.850
2.117	12.452	7.835

Ordinary one-way ANOVA
ANOVA results

1	Table Analyzed	Figure 4E				
2	Data sets analyzed	A-C				
3						
4	**ANOVA summary**					
5	F	107.4				
6	P value	<0.0001				
7	P value summary	****				
8	Significant diff. among means (P < 0.05)'	Yes				
9	R squared	0.9471				
10						
11	**Brown-Forsythe test**					
12	F (DFn, DFd)	2.461 (2, 12)				
13	P value	0.1272				
14	P value summary	ns				
15	Are SDs significantly different (P < 0.05)?	No				
16						
17	**Bartlett's test**					
18	Bartlett's statistic (corrected)	8.986				
19	P value	0.0112				
20	P value summary	*				
21	Are SDs significantly different (P < 0.05)?	Yes				
22						
23	**ANOVA table**	**SS**	**DF**	**MS**	**F (DFn, DFd)**	**P value**
24	Treatment (between columns)	301.6	2	150.8	F (2, 12) = 107.4	P<0.0001
25	Residual (within columns)	16.85	12	1.404		
26	Total	318.5	14			
27						
28	**Data summary**					
29	Number of treatments (columns)	3				
30	Number of values (total)	15				

Ordinary one-way ANOVA
Multiple comparisons

1	Number of families	1							
2	Number of comparisons per family	3							
3	Alpha	0.05							
4									
5	**Tukey's multiple comparisons test**	**Mean Diff**	**95.00% CI of diff.**	**Below threshold?**	**Summary**	**Adjusted P Value**			
6	Normal vs. OVA	-10.94	-12.94 to -8.937	Yes	****	<0.0001	A-B		
7	Normal vs. OVA+ROS	-6.351	-8.351 to -4.352	Yes	****	<0.0001	A-C		
8	OVA vs. OVA+ROS	4.585	2.586 to 6.585	Yes	***	0.0001	B-C		
9									
10	**Test details**	**Mean 1**	**Mean 2**	**Mean Diff.**	**SE of diff.**	**n1**	**n2**	**q**	**DF**
11	Normal vs. OVA	1.995	12.93	-10.94	0.7494	5	5	20.64	12
12	Normal vs. OVA+ROS	1.995	8.347	-6.351	0.7494	5	5	11.99	12
13	OVA vs. OVA+ROS	12.93	8.347	4.585	0.7494	5	5	8.652	12

Figure 4F

	Group A	Group B	Group C
	Normal	OVA	OVA+ROS
1	3.493	12.510	11.600
2	3.305	19.838	9.984
3	2.622	15.479	9.457
4	2.607	13.245	9.317
5	2.452	18.659	8.450

	Ordinary one-way ANOVA<br>ANOVA results					
10						
11	**Brown-Forsythe test**					
12	F (DFn, DFd)	5.822 (2, 12)				
13	P value	0.0171				
14	P value summary	*				
15	Are SDs significantly different (P < 0.05)'	Yes				
16						
17	**Bartlett's test**					
18	Bartlett's statistic (corrected)	10.90				
19	P value	0.0043				
20	P value summary	**				
21	Are SDs significantly different (P < 0.05)'	Yes				
22						
23	**ANOVA table**	**SS**	**DF**	**MS**	**F (DFn, DFd**	**P value**
24	Treatment (between columns)	426.2	2	213.1	F (2, 12) = 5	P<0.0001
25	Residual (within columns)	48.15	12	4.012		
26	Total	474.3	14			
27						
28	**Data summary**					
29	Number of treatments (columns)	3				
30	Number of values (total)	15				

	Ordinary one-way ANOVA<br>Multiple comparisons								
1	Number of families	1							
2	Number of comparisons per family	3							
3	Alpha	0.05							
4									
5	**Tukey's multiple comparisons test**	**Mean Diff**	**95.00% CI of diff.**	**Below threshold?**	**Summary**	**Adjusted P Value**			
6	Normal vs. OVA	-13.05	-16.43 to -9.671	Yes	****	<0.0001	A-B		
7	Normal vs. OVA+ROS	-6.866	-10.25 to -3.486	Yes	***	0.0004	A-C		
8	OVA vs. OVA+ROS	6.185	2.805 to 9.564	Yes	**	0.0010	B-C		
9									
10	**Test details**	**Mean 1**	**Mean 2**	**Mean Diff.**	**SE of diff.**	**n1**	**n2**	**q**	**DF**
11	Normal vs. OVA	2.896	15.95	-13.05	1.267	5	5	14.57	12
12	Normal vs. OVA+ROS	2.896	9.762	-6.866	1.267	5	5	7.664	12
13	OVA vs. OVA+ROS	15.95	9.762	6.185	1.267	5	5	6.904	12

Figure 4G

	Group A	Group B	Group C
	Normal	OVA	OVA+ROS
1	24.416	169.027	120.396
2	31.968	140.119	100.639
3	42.015	148.775	142.351
4	31.128	136.116	99.640
5	28.835	148.913	124.510

	Ordinary one-way ANOVA<br>ANOVA results					
1	Table Analyzed	Figure 4G				
2	Data sets analyzed	A-C				
3						
4	**ANOVA summary**					
5	F	105.2				
6	P value	<0.0001				
7	P value summary	****				
8	Significant diff. among means (P < 0.05)'	Yes				
9	R squared	0.9460				
10						
11	**Brown-Forsythe test**					
12	F (DFn, DFd)	1.552 (2, 12)				
13	P value	0.2514				
14	P value summary	ns				
15	Are SDs significantly different (P < 0.05)'	No				
16						
17	**Bartlett's test**					
18	Bartlett's statistic (corrected)	3.219				
19	P value	0.2000				
20	P value summary	ns				
21	Are SDs significantly different (P < 0.05)'	No				
22						
23	**ANOVA table**	**SS**	**DF**	**MS**	**F (DFn, DFd)**	**P value**
24	Treatment (between columns)	36672	2	18336	F (2, 12) = 105.2	P<0.0001
25	Residual (within columns)	2092	12	174.3		
26	Total	38764	14			
27						
28	**Data summary**					
29	Number of treatments (columns)	3				
30	Number of values (total)	15				

	Ordinary one-way ANOVA<br>Multiple comparisons									
1	Number of families	1								
2	Number of comparisons per family	3								
3	Alpha	0.05								
4										
5	**Tukey's multiple comparisons test**	**Mean Diff.**	**95.00% CI of diff.**	**Below threshold?**	**Summary**	**Adjusted P Value**				
6	Normal vs. OVA	-116.9	-139.2 to -94.64	Yes	****	<0.0001	A-B			
7	Normal vs. OVA+ROS	-85.83	-108.1 to -63.56	Yes	****	<0.0001	A-C			
8	OVA vs. OVA+ROS	31.08	8.807 to 53.36	Yes	**	0.0076	B-C			
9										
10	**Test details**	**Mean 1**	**Mean 2**	**Mean Diff.**	**SE of diff.**	**n1**	**n2**	**q**	**DF**	
11	Normal vs. OVA	31.67	148.6	-116.9	8.350	5	5	19.80	12	
12	Normal vs. OVA+ROS	31.67	117.5	-85.83	8.350	5	5	14.54	12	
13	OVA vs. OVA+ROS	148.6	117.5	31.08	8.350	5	5	5.264	12	

Figure 4H

	Group A	Group B	Group C
	Normal	OVA	OVA+ROS
1	2.918	13.095	10.397
2	3.393	13.253	10.068
3	3.175	13.710	8.509
4	3.233	17.975	9.181
5	2.256	15.759	10.588

	Ordinary one-way ANOVA<br>ANOVA results					
1	Table Analyzed	Figure 4H				
2	Data sets analyzed	A-C				
3						
4	**ANOVA summary**					
5	F	97.86				
6	P value	<0.0001				
7	P value summary	****				
8	Significant diff. among means (P < 0.05)?	Yes				
9	R squared	0.9422				
10						
11	**Brown-Forsythe test**					
12	F (DFn, DFd)	1.578 (2, 12)				
13	P value	0.2464				
14	P value summary	ns				
15	Are SDs significantly different (P < 0.05)?	No				
16						
17	**Bartlett's test**					
18	Bartlett's statistic (corrected)	7.651				
19	P value	0.0218				
20	P value summary	*				
21	Are SDs significantly different (P < 0.05)?	Yes				
22						
23	**ANOVA table**	**SS**	**DF**	**MS**	**F (DFn, DFd)**	**P value**
24	Treatment (between columns)	348.5	2	174.2	F (2, 12) = 97.86	P<0.0001
25	Residual (within columns)	21.37	12	1.780		
26	Total	369.8	14			
27						
28	**Data summary**					
29	Number of treatments (columns)	3				
30	Number of values (total)	15				

	Ordinary one-way ANOVA<br>Multiple comparisons								
1	Number of families	1							
2	Number of comparisons per family	3							
3	Alpha	0.05							
4									
5	**Tukey's multiple comparisons test**	**Mean Diff.**	**95.00% CI of diff.**	**Below threshold?**	**Summary**	**Adjusted P Value**			
6	Normal vs. OVA	-11.76	-14.01 to -9.512	Yes	****	<0.0001	A-B		
7	Normal vs. OVA+ROS	-6.754	-9.005 to -4.502	Yes	****	<0.0001	A-C		
8	OVA vs. OVA+ROS	5.010	2.758 to 7.261	Yes	***	0.0002	B-C		
9									
10	**Test details**	**Mean 1**	**Mean 2**	**Mean Diff.**	**SE of diff.**	**n1**	**n2**	**q**	**DF**
11	Normal vs. OVA	2.995	14.76	-11.76	0.8439	5	5	19.71	12
12	Normal vs. OVA+ROS	2.995	9.749	-6.754	0.8439	5	5	11.32	12
13	OVA vs. OVA+ROS	14.76	9.749	5.010	0.8439	5	5	8.395	12

Figure 5A

	Group A	Group B	Group C
	Normal	OVA	OVA+ROS
1	257.268	1024.914	828.895
2	330.564	1210.087	811.176
3	302.266	1011.605	696.343
4	227.163	1445.076	881.858
5	285.071	870.464	915.286

	Ordinary one-way ANOVA<br>ANOVA results					
1	Table Analyzed	Figure 5A				
2	Data sets analyzed	A-C				
3						
4	**ANOVA summary**					
5	F	46.40				
6	P value	<0.0001				
7	P value summary	****				
8	Significant diff. among means (P < 0.05)?	Yes				
9	R squared	0.8855				
10						
11	**Brown-Forsythe test**					
12	F (DFn, DFd)	2.000 (2, 12)				
13	P value	0.1780				
14	P value summary	ns				
15	Are SDs significantly different (P < 0.05)?	No				
16						
17	**Bartlett's test**					
18	Bartlett's statistic (corrected)	9.218				
19	P value	0.0100				
20	P value summary	**				
21	Are SDs significantly different (P < 0.05)?	Yes				
22						
23	**ANOVA table**	**SS**	**DF**	**MS**	**F (DFn, DFd)**	**P value**
24	Treatment (between columns)	1786967	2	893484	F (2, 12) = 46.40	P<0.0001
25	Residual (within columns)	231077	12	19256		
26	Total	2018044	14			
27						
28	**Data summary**					
29	Number of treatments (columns)	3				
30	Number of values (total)	15				

	Ordinary one-way ANOVA<br>Multiple comparisons								
1	Number of families	1							
2	Number of comparisons per family	3							
3	Alpha	0.05							
4									
5	**Tukey's multiple comparisons test**	**Mean Diff.**	**95.00% CI of diff.**	**Below threshold?**	**Summary**	**Adjusted P Valu**			
6	Normal vs. OVA	-832.0	-1066 to -597.8	Yes	****	<0.0001	A-B		
7	Normal vs. OVA+ROS	-546.2	-780.4 to -312.1	Yes	***	0.0001	A-C		
8	OVA vs. OVA+ROS	285.7	51.57 to 519.9	Yes	*	0.0175	B-C		
9									
10	**Test details**	**Mean 1**	**Mean 2**	**Mean Diff.**	**SE of diff.**	**n1**	**n2**	**q**	**DF**
11	Normal vs. OVA	280.5	1112	-832.0	87.76	5	5	13.41	12
12	Normal vs. OVA+ROS	280.5	826.7	-546.2	87.76	5	5	8.802	12
13	OVA vs. OVA+ROS	1112	826.7	285.7	87.76	5	5	4.604	12

Figure 5B

	Group A	Group B	Group C
	Normal	OVA	OVA+ROS
1	33.896	70.091	42.683
2	28.824	66.600	54.753
3	22.569	71.432	44.036
4	18.591	67.821	56.311
5	29.337	64.276	53.387

	Ordinary one-way ANOVA<br>ANOVA results					
1	Table Analyzed	Figure 5B				
2	Data sets analyzed	A-C				
3						
4	**ANOVA summary**					
5	F	75.94				
6	P value	<0.0001				
7	P value summary	****				
8	Significant diff. among means (P < 0.05)?	Yes				
9	R squared	0.9268				
10						
11	**Brown-Forsythe test**					
12	F (DFn, DFd)	0.7355 (2, 12)				
13	P value	0.4997				
14	P value summary	ns				
15	Are SDs significantly different (P < 0.05)?	No				
16						
17	**Bartlett's test**					
18	Bartlett's statistic (corrected)	2.362				
19	P value	0.3070				
20	P value summary	ns				
21	Are SDs significantly different (P < 0.05)?	No				
22						
23	**ANOVA table**	**SS**	**DF**	**MS**	**F (DFn, DFd)**	**P value**
24	Treatment (between columns)	4313	2	2156	F (2, 12) = 75.94	P<0.0001
25	Residual (within columns)	340.8	12	28.40		
26	Total	4654	14			
27						
28	**Data summary**					
29	Number of treatments (columns)	3				
30	Number of values (total)	15				

	Ordinary one-way ANOVA<br>Multiple comparisons								
1	Number of families	1							
2	Number of comparisons per family	3							
3	Alpha	0.05							
4									
5	**Tukey's multiple comparisons test**	**Mean Diff.**	**95.00% CI of diff.**	**Below threshold?**	**Summary**	**Adjusted P Value**			
6	Normal vs. OVA	-41.40	-50.39 to -32.41	Yes	****	<0.0001	A-B		
7	Normal vs. OVA+ROS	-23.59	-32.58 to -14.60	Yes	****	<0.0001	A-C		
8	OVA vs. OVA+ROS	17.81	8.818 to 26.80	Yes	***	0.0005	B-C		
9									
10	**Test details**	**Mean 1**	**Mean 2**	**Mean Diff.**	**SE of diff.**	**n1**	**n2**	**q**	**DF**
11	Normal vs. OVA	26.64	68.04	-41.40	3.370	5	5	17.37	12
12	Normal vs. OVA+ROS	26.64	50.23	-23.59	3.370	5	5	9.899	12
13	OVA vs. OVA+ROS	68.04	50.23	17.81	3.370	5	5	7.473	12

Figure 5C

	Group A	Group B	Group C
	Normal	OVA	OVA+ROS
1	61.626	102.206	95.932
2	73.726	146.669	104.819
3	69.217	147.852	122.814
4	51.939	132.294	115.032
5	58.837	166.144	84.012

	Ordinary one-way ANOVA<br>ANOVA results					
1	Table Analyzed	Figure 5C				
2	Data sets analyzed	A-C				
3						
4	**ANOVA summary**					
5	F	24.75				
6	P value	<0.0001				
7	P value summary	****				
8	Significant diff. among means (P < 0.05)?	Yes				
9	R squared	0.8049				
10						
11	**Brown-Forsythe test**					
12	F (DFn, DFd)	0.8076 (2, 12)				
13	P value	0.4688				
14	P value summary	ns				
15	Are SDs significantly different (P < 0.05)?	No				
16						
17	**Bartlett's test**					
18	Bartlett's statistic (corrected)	3.349				
19	P value	0.1874				
20	P value summary	ns				
21	Are SDs significantly different (P < 0.05)?	No				
22						
23	**ANOVA table**	**SS**	**DF**	**MS**	**F (DFn, DFd)**	**P value**
24	Treatment (between columns)	14466	2	7233	F (2, 12) = 24.75	P<0.0001
25	Residual (within columns)	3508	12	292.3		
26	Total	17974	14			
27						
28	**Data summary**					
29	Number of treatments (columns)	3				
30	Number of values (total)	15				

	Ordinary one-way ANOVA<br>Multiple comparisons								
1	Number of families	1							
2	Number of comparisons per family	3							
3	Alpha	0.05							
4									
5	**Tukey's multiple comparisons test**	**Mean Diff.**	**95.00% CI of diff.**	**Below threshold?**	**Summary**	**Adjusted P Value**			
6	Normal vs. OVA	-75.96	-104.8 to -47.12	Yes	****	<0.0001	A-B		
7	Normal vs. OVA+ROS	-41.45	-70.30 to -12.61	Yes	**	0.0062	A-C		
8	OVA vs. OVA+ROS	34.51	5.664 to 63.36	Yes	*	0.0197	B-C		
9									
10	**Test details**	**Mean 1**	**Mean 2**	**Mean Diff.**	**SE of diff.**	**n1**	**n2**	**q**	**DF**
11	Normal vs. OVA	63.07	139.0	-75.96	10.81	5	5	9.935	12
12	Normal vs. OVA+ROS	63.07	104.5	-41.45	10.81	5	5	5.422	12
13	OVA vs. OVA+ROS	139.0	104.5	34.51	10.81	5	5	4.514	12

Figure 5D

	Group A	Group B	Group C
	Normal	OVA	OVA+ROS
1	108.815	258.497	175.357
2	127.645	227.347	170.206
3	115.154	175.809	163.328
4	109.261	194.641	166.982
5	135.039	237.850	141.384

	Ordinary one-way ANOVA<br>ANOVA results					
1	Table Analyzed	Figure 5D				
2	Data sets analyzed	A-C				
3						
4	**ANOVA summary**					
5	F	26.35				
6	P value	<0.0001				
7	P value summary	****				
8	Significant diff. among means (P < 0.05)'	Yes				
9	R squared	0.8145				
10						
11	**Brown-Forsythe test**					
12	F (DFn, DFd)	2.430 (2, 12)				
13	P value	0.1300				
14	P value summary	ns				
15	Are SDs significantly different (P < 0.05)?	No				
16						
17	**Bartlett's test**					
18	Bartlett's statistic (corrected)	5.053				
19	P value	0.0799				
20	P value summary	ns				
21	Are SDs significantly different (P < 0.05)?	No				
22						
23	**ANOVA table**	**SS**	**DF**	**MS**	**F (DFn, DFd)**	**P value**
24	Treatment (between columns)	24926	2	12463	F (2, 12) = 26.35	P<0.0001
25	Residual (within columns)	5676	12	473.0		
26	Total	30602	14			
27						
28	**Data summary**					
29	Number of treatments (columns)	3				
30	Number of values (total)	15				

	Ordinary one-way ANOVA<br>Multiple comparisons								
1	Number of families	1							
2	Number of comparisons per family	3							
3	Alpha	0.05							
4									
5	**Tukey's multiple comparisons test**	**Mean Diff.**	**95.00% CI of diff.**	**Below threshold?**	**Summary**	**Adjusted P Value**			
6	Normal vs. OVA	-99.65	-136.3 to -62.95	Yes	****	<0.0001	A-B		
7	Normal vs. OVA+ROS	-44.27	-80.96 to -7.573	Yes	*	0.0187	A-C		
8	OVA vs. OVA+ROS	55.38	18.68 to 92.07	Yes	**	0.0044	B-C		
9									
10	**Test details**	**Mean 1**	**Mean 2**	**Mean Diff.**	**SE of diff.**	**n1**	**n2**	**q**	**DF**
11	Normal vs. OVA	119.2	218.8	-99.65	13.75	5	5	10.25	12
12	Normal vs. OVA+ROS	119.2	163.5	-44.27	13.75	5	5	4.552	12
13	OVA vs. OVA+ROS	218.8	163.5	55.38	13.75	5	5	5.694	12

Figure 6B

	Group A	Group B	Group C
	Normal	OVA	OVA+ROS
1	0.035	0.018	0.093
2	0.028	0.018	0.091
3	0.036	0.020	0.086
4	0.036	0.021	0.105
5	0.027	0.018	0.091

	Ordinary one-way ANOVA<br>ANOVA results					
1	Table Analyzed	Figure 6B				
2	Data sets analyzed	A-C				
3						
4	**ANOVA summary**					
5	F	323.5				
6	P value	<0.0001				
7	P value summary	****				
8	Significant diff. among means (P < 0.05)?	Yes				
9	R squared	0.9818				
10						
11	**Brown-Forsythe test**					
12	F (DFn, DFd)	0.8238 (2, 12)				
13	P value	0.4621				
14	P value summary	ns				
15	Are SDs significantly different (P < 0.05)?	No				
16						
17	**Bartlett's test**					
18	Bartlett's statistic (corrected)	6.967				
19	P value	0.0307				
20	P value summary	*				
21	Are SDs significantly different (P < 0.05)?	Yes				
22						
23	**ANOVA table**	**SS**	**DF**	**MS**	**F (DFn, DFd)**	**P value**
24	Treatment (between columns)	0.01564	2	0.007818	F (2, 12) = 323.5	P<0.0001
25	Residual (within columns)	0.0002900	12	2.417e-005		
26	Total	0.01593	14			
27						
28	**Data summary**					
29	Number of treatments (columns)	3				
30	Number of values (total)	15				

	Ordinary one-way ANOVA<br>Multiple comparisons								
1	Number of families	1							
2	Number of comparisons per family	3							
3	Alpha	0.05							
4									
5	**Tukey's multiple comparisons test**	**Mean Diff.**	**95.00% CI of diff.**	**Below threshold?**	**Summary**	**Adjusted P Value**			
6	Normal vs. OVA	0.01340	0.005105 to 0.02169	Yes	**	0.0027	A-B		
7	Normal vs. OVA+ROS	-0.06080	-0.06909 to -0.05251	Yes	****	<0.0001	A-C		
8	OVA vs. OVA+ROS	-0.07420	-0.08249 to -0.06591	Yes	****	<0.0001	B-C		
9									
10	**Test details**	**Mean 1**	**Mean 2**	**Mean Diff.**	**SE of diff.**	**n1**	**n2**	**q**	**DF**
11	Normal vs. OVA	0.03240	0.01900	0.01340	0.003109	5	5	6.095	12
12	Normal vs. OVA+ROS	0.03240	0.09320	-0.06080	0.003109	5	5	27.66	12
13	OVA vs. OVA+ROS	0.01900	0.09320	-0.07420	0.003109	5	5	33.75	12

Figure 6C

Group A	Group B	Group C
Normal	OVA	OVA+ROS
0.839	0.104	0.558
0.998	0.107	0.918
0.649	0.091	0.809

Ordinary one-way ANOVA
ANOVA results

Table Analyzed	Figure 6C				
Data sets analyzed	A-C				
**ANOVA summary**					
F	22.53				
P value	0.0016				
P value summary	**				
Significant diff. among means (P < 0.05)'	Yes				
R squared	0.8825				
**Brown-Forsythe test**					
F (DFn, DFd)	1.455 (2, 6)				
P value	0.3054				
P value summary	ns				
Are SDs significantly different (P < 0.05)?	No				
**Bartlett's test**					
Bartlett's statistic (corrected)					
P value					
P value summary					
Are SDs significantly different (P < 0.05)?					
**ANOVA table**	**SS**	**DF**	**MS**	**F (DFn, DFd)**	**P value**
Treatment (between columns)	0.9714	2	0.4857	F (2, 6) = 22.53	P=0.0016
Residual (within columns)	0.1294	6	0.02156		
Total	1.101	8			
**Data summary**					
Number of treatments (columns)	3				
Number of values (total)	9				

Ordinary one-way ANOVA
Multiple comparisons

Number of families	1							
Number of comparisons per family	3							
Alpha	0.05							
**Tukey's multiple comparisons test**	**Mean Diff.**	**95.00% CI of diff.**	**Below threshold?**	**Summary**	**Adjusted P Value**			
Normal vs. OVA	0.7280	0.3601 to 1.096	Yes	**	0.0022	A-B		
Normal vs. OVA+ROS	0.06700	-0.3009 to 0.4349	No	ns	0.8460	A-C		
OVA vs. OVA+ROS	-0.6610	-1.029 to -0.2931	Yes	**	0.0036	B-C		
**Test details**	**Mean 1**	**Mean 2**	**Mean Diff.**	**SE of diff.**	**n1**	**n2**	**q**	**DF**
Normal vs. OVA	0.8287	0.1007	0.7280	0.1199	3	3	8.587	6
Normal vs. OVA+ROS	0.8287	0.7617	0.06700	0.1199	3	3	0.7903	6
OVA vs. OVA+ROS	0.1007	0.7617	-0.6610	0.1199	3	3	7.797	6

Figure 6F

	Group A	Group B	Group C
	Normal	OVA	OVA+ROS
1	60.221	189.779	78.458
2	57.966	139.548	69.634
3	55.064	110.414	89.222
4	45.028	125.303	61.994
5	66.512	114.619	84.797

	Ordinary one-way ANOVA<br>ANOVA results					
1	Table Analyzed	Figure 6F				
2	Data sets analyzed	A-C				
3						
4	**ANOVA summary**					
5	F	20.79				
6	P value	0.0001				
7	P value summary	***				
8	Significant diff. among means (P < 0.05)	Yes				
9	R squared	0.7760				
10						
11	**Brown-Forsythe test**					
12	F (DFn, DFd)	1.456 (2, 12)				
13	P value	0.2716				
14	P value summary	ns				
15	Are SDs significantly different (P < 0.05)	No				
16						
17	**Bartlett's test**					
18	Bartlett's statistic (corrected)	7.693				
19	P value	0.0214				
20	P value summary	*				
21	Are SDs significantly different (P < 0.05)	Yes				
22						
23	**ANOVA table**	**SS**	**DF**	**MS**	**F (DFn, DFd)**	**P value**
24	Treatment (between columns)	16876	2	8438	F (2, 12) = 20.79	P=0.0001
25	Residual (within columns)	4871	12	405.9		
26	Total	21748	14			
27						
28	**Data summary**					
29	Number of treatments (columns)	3				
30	Number of values (total)	15				

	Ordinary one-way ANOVA<br>Multiple comparisons								
1	Number of families	1							
2	Number of comparisons per family	3							
3	Alpha	0.05							
4									
5	**Tukey's multiple comparisons test**	**Mean Diff.**	**95.00% CI of diff.**	**Below threshold?**	**Summary**	**Adjusted P Value**			
6	Normal vs. OVA	-78.97	-113.0 to -44.98	Yes	***	0.0001	A-B		
7	Normal vs. OVA+ROS	-19.86	-53.86 to 14.13	No	ns	0.3000	A-C		
8	OVA vs. OVA+ROS	59.11	25.12 to 93.11	Yes	**	0.0015	B-C		
9									
10	**Test details**	**Mean 1**	**Mean 2**	**Mean Diff.**	**SE of diff.**	**n1**	**n2**	**q**	**DF**
11	Normal vs. OVA	56.96	135.9	-78.97	12.74	5	5	8.765	12
12	Normal vs. OVA+ROS	56.96	76.82	-19.86	12.74	5	5	2.204	12
13	OVA vs. OVA+ROS	135.9	76.82	59.11	12.74	5	5	6.560	12

Figure 6G

	Group A	Group B	Group C
	Normal	OVA	OVA+ROS
1	1.038	2.393	1.889
2	1.004	2.704	2.177
3	0.935	3.179	1.891
4	0.860	2.281	2.168
5	0.775	2.616	1.954

	Ordinary one-way ANOVA<br>ANOVA results					
1	Table Analyzed	Figure 6G				
2	Data sets analyzed	A-C				
3						
4	**ANOVA summary**					
5	F	73.29				
6	P value	<0.0001				
7	P value summary	****				
8	Significant diff. among means (P < 0.05)?	Yes				
9	R squared	0.9243				
10						
11	**Brown-Forsythe test**					
12	F (DFn, DFd)	1.749 (2, 12)				
13	P value	0.2155				
14	P value summary	ns				
15	Are SDs significantly different (P < 0.05)?	No				
16						
17	**Bartlett's test**					
18	Bartlett's statistic (corrected)	5.487				
19	P value	0.0643				
20	P value summary	ns				
21	Are SDs significantly different (P < 0.05)?	No				
22						
23	**ANOVA table**	**SS**	**DF**	**MS**	**F (DFn, DFd)**	**P value**
24	Treatment (between columns)	7.517	2	3.758	F (2, 12) = 73.29	P<0.0001
25	Residual (within columns)	0.6154	12	0.05128		
26	Total	8.132	14			
27						
28	**Data summary**					
29	Number of treatments (columns)	3				
30	Number of values (total)	15				

	Ordinary one-way ANOVA<br>Multiple comparisons								
1	Number of families	1							
2	Number of comparisons per family	3							
3	Alpha	0.05							
4									
5	**Tukey's multiple comparisons test**	**Mean Diff.**	**95.00% CI of diff.**	**Below threshold?**	**Summary**	**Adjusted P Value**			
6	Normal vs. OVA	-1.712	-2.094 to -1.330	Yes	****	<0.0001	A-B		
7	Normal vs. OVA+ROS	-1.093	-1.475 to -0.7113	Yes	****	<0.0001	A-C		
8	OVA vs. OVA+ROS	0.6188	0.2367 to 1.001	Yes	**	0.0026	B-C		
9									
10	**Test details**	**Mean 1**	**Mean 2**	**Mean Diff.**	**SE of diff.**	**n1**	**n2**	**q**	**DF**
11	Normal vs. OVA	0.9224	2.635	-1.712	0.1432	5	5	16.91	12
12	Normal vs. OVA+ROS	0.9224	2.016	-1.093	0.1432	5	5	10.80	12
13	OVA vs. OVA+ROS	2.635	2.016	0.6188	0.1432	5	5	6.110	12

Figure 6H

	Group A	Group B	Group C
	Normal	OVA	OVA+ROS
1	0.010	0.055	0.041
2	0.009	0.073	0.042
3	0.008	0.054	0.043

Ordinary one-way ANOVA
ANOVA results

1	Table Analyzed	Figure 6H				
2	Data sets analyzed	A-C				
3						
4	**ANOVA summary**					
5	F	52.95				
6	P value	0.0002				
7	P value summary	***				
8	Significant diff. among means (P < 0.05)'	Yes				
9	R squared	0.9464				
10						
11	**Brown-Forsythe test**					
12	F (DFn, DFd)	0.9353 (2, 6)				
13	P value	0.4430				
14	P value summary	ns				
15	Are SDs significantly different (P < 0.05)'	No				
16						
17	**Bartlett's test**					
18	Bartlett's statistic (corrected)					
19	P value					
20	P value summary					
21	Are SDs significantly different (P < 0.05)'					
22						
23	**ANOVA table**	**SS**	**DF**	**MS**	**F (DFn, DFd)**	**P value**
24	Treatment (between columns)	0.004107	2	0.002053	F (2, 6) = 52.95	P=0.0002
25	Residual (within columns)	0.0002327	6	3.878e-005		
26	Total	0.004340	8			
27						
28	**Data summary**					
29	Number of treatments (columns)	3				
30	Number of values (total)	9				

Ordinary one-way ANOVA
Multiple comparisons

1	Number of families	1							
2	Number of comparisons per famil	3							
3	Alpha	0.05							
4									
5	**Tukey's multiple comparisons t**	**Mean Diff.**	**95.00% CI of diff.**	**Below threshold?**	**Summary**	**Adjusted P Value**			
6	Normal vs. OVA	-0.05167	-0.06727 to -0.03607	Yes	***	0.0001	A-B		
7	Normal vs. OVA+ROS	-0.03300	-0.04860 to -0.01740	Yes	**	0.0015	A-C		
8	OVA vs. OVA+ROS	0.01867	0.003066 to 0.03427	Yes	*	0.0243	B-C		
9									
10	**Test details**	**Mean 1**	**Mean 2**	**Mean Diff.**	**SE of diff.**	**n1**	**n2**	**q**	**DF**
11	Normal vs. OVA	0.009000	0.06067	-0.05167	0.005084	3	3	14.37	6
12	Normal vs. OVA+ROS	0.009000	0.04200	-0.03300	0.005084	3	3	9.179	6
13	OVA vs. OVA+ROS	0.06067	0.04200	0.01867	0.005084	3	3	5.192	6
